# Machine learning to understand risks for severe COVID-19 outcomes: a retrospective cohort study of immune-mediated inflammatory diseases, immunomodulatory medications, and comorbidities in a large US health-care system

**DOI:** 10.1016/S2589-7500(24)00021-9

**Published:** 2024-05

**Authors:** Qi Wei, Philip J Mease, Michael Chiorean, Lulu Iles-Shih, Wanessa F Matos, Andrew Baumgartner, Sevda Molani, Yeon Mi Hwang, Basazin Belhu, Alexandra Ralevski, Jennifer Hadlock

**Affiliations:** **Institute for Systems Biology, Seattle, WA, USA** (Q Wei PhD, W F Matos MD, A Baumgartner PhD, S Molani PhD, Y Mi Hwang MSc, B Belhu BSc, A Ralevski PhD, J Hadlock MD)**; Providence St Joseph Health–Swedish Medical Center, Seattle, WA, USA** (Prof P J Mease MD)**; Digestive Health Institute, Swedish Medical Center, Seattle, WA, USA** (M Chiorean MD, L Iles-Shih MD)**; Biomedical Informatics and Medical Education, University of Washington, Seattle, WA, USA** (J Hadlock)

## Abstract

**Background:**

In the context of immune-mediated inflammatory diseases (IMIDs), COVID-19 outcomes are incompletely understood and vary considerably depending on the patient population studied. We aimed to analyse severe COVID-19 outcomes and to investigate the effects of the pandemic time period and the risks associated with individual IMIDs, classes of immunomodulatory medications (IMMs), chronic comorbidities, and COVID-19 vaccination status.

**Methods:**

In this retrospective cohort study, clinical data were derived from the electronic health records of an integrated health-care system serving patients in 51 hospitals and 1085 clinics across seven US states (Providence St Joseph Health). Data were observed for patients (no age restriction) with one or more IMID and for unmatched controls without IMIDs. COVID-19 was identified with a positive nucleic acid amplification test result for SARS-CoV-2. Two timeframes were analysed: March 1, 2020–Dec 25, 2021 (pre-omicron period), and Dec 26, 2021–Aug 30, 2022 (omicron-predominant period). Primary outcomes were hospitalisation, mechanical ventilation, and mortality in patients with COVID-19. Factors, including IMID diagnoses, comorbidities, long-term use of IMMs, and COVID-19 vaccination status, were analysed with multivariable logistic regression (LR) and extreme gradient boosting (XGB).

**Findings:**

Of 2 167 656 patients tested for SARS-CoV-2, 290 855 (13·4%) had confirmed COVID-19: 15 397 (5·3%) patients with IMIDs and 275 458 (94·7%) without IMIDs. In the pre-omicron period, 169 993 (11·2%) of 1 517 295 people who were tested for COVID-19 tested positive, of whom 23 330 (13·7%) were hospitalised, 1072 (0·6%) received mechanical ventilation, and 5294 (3·1%) died. Compared with controls, patients with IMIDs and COVID-19 had higher rates of hospitalisation (1176 [14·6%] *vs* 22 154 [13·7%]; p=0·024) and mortality (314 [3·9%] *vs* 4980 [3·1%]; p<0·0001). In the omicron-predominant period, 120 862 (18·6%) of 650 361 patients tested positive for COVID-19, of whom 14 504 (12·0%) were hospitalised, 567 (0·5%) received mechanical ventilation, and 2001 (1·7%) died. Compared with controls, patients with IMIDs and COVID-19 (7327 [17·3%] of 42 249) had higher rates of hospitalisation (13 422 [11·8%] *vs* 1082 [14·8%]; p<0·0001) and mortality (1814 [1·6%] *vs* 187 [2·6%]; p<0·0001). Age was a risk factor for worse outcomes (adjusted odds ratio [OR] from 2·1 [95% CI 2·0–2·1]; p<0·0001 to 3·0 [2·9–3·0]; p<0·0001), whereas COVID-19 vaccination (from 0·082 [0·080–0·085]; p<0·0001 to 0·52 [0·50–0·53]; p<0·0001) and booster vaccination (from 2·1 [2·0–2·2]; p<0·0001 to 3·0 [2·9–3·0]; p<0·0001) status were associated with better outcomes. Seven chronic comorbidities were significant risk factors during both time periods for all three outcomes: atrial fibrillation, coronary artery disease, heart failure, chronic kidney disease, chronic obstructive pulmonary disease, chronic liver disease, and cancer. Two IMIDs, asthma (adjusted OR from 0·33 [0·32–0·34]; p<0·0001 to 0·49 [0·48–0·51]; p<0·0001) and psoriasis (from 0·52 [0·48–0·56] to 0·80 [0·74–0·87]; p<0·0001), were associated with a reduced risk of severe outcomes. IMID diagnoses did not appear to be significant risk factors themselves, but results were limited by small sample size, and vasculitis had high feature importance in LR. IMMs did not appear to be significant, but less frequently used IMMs were limited by sample size. XGB outperformed LR, with the area under the receiver operating characteristic curve for models across different time periods and outcomes ranging from 0·77 to 0·92.

**Interpretation:**

Our results suggest that age, chronic comorbidities, and not being fully vaccinated might be greater risk factors for severe COVID-19 outcomes in patients with IMIDs than the use of IMMs or the IMIDs themselves. Overall, there is a need to take age and comorbidities into consideration when developing COVID-19 guidelines for patients with IMIDs. Further research is needed for specific IMIDs (including IMID severity at the time of SARS-CoV-2 infection) and IMMs (considering dosage and timing before a patient’s first COVID-19 infection).

## Introduction

COVID-19 remains a challenge worldwide, with more than 7·0 million reported deaths from the disease as of Feb 28, 2024.^1^ Given the variability in the course and outcomes of COVID-19 and its relationship with the immunological system, understanding outcomes in patients with immune-mediated inflammatory diseases (IMIDs) is essential.

IMIDs are a set of clinically diverse conditions characterised by immune dysregulation, chronic inflammation, and potential organ damage. IMIDs include autoimmune diseases, such as rheumatoid arthritis and multiple sclerosis, as well as inflammatory conditions, including allergic asthma. Given established and potential COVID-19 risk factors, individuals with IMIDs are of particular interest for risk analysis due to the complexity of the diseases.^2–5^ This patient population has an increased rate of severe COVID-19 outcomes; however, reasons why remain unclear. Potential reasons include immune dysregulation, the use of immunomodulatory medications (IMMs), and associated chronic comorbidities. Furthermore, comorbidities associated with severe COVID-19 outcomes, including heart disease and diabetes, are higher among patients with IMIDs than in the general population.^6^ IMMs for IMIDs could theoretically foster viral replication, which might not only be detrimental in the early stages of COVID-19, but also reduce the systemic inflammatory response syndrome associated with organ damage, morbidity, and mortality.^7^ Additionally, patients can have multiple IMIDs or be taking multiple IMMs. Furthermore, allergic asthma has been shown to be associated with a reduced susceptibility to severe COVID-19 outcomes, leading to new insights on the intrinsic factors modulating intracellular viral load and and cell-to-cell transmission.^5^

Health outcomes must also be considered in the context of changes over the course of the pandemic, including SARS-CoV-2 variants, increased access to COVID-19 vaccination, and changes in the standard of care for COVID-19 treatment. Previous research suggests that, as well as advanced age, specific chronic comorbidities have been associated with an increased risk of hospitalisation and death among patients with COVID-19 and IMIDs, including diabetes, chronic kidney disease, chronic obstructive pulmonary disease (COPD), cardiovascular disease, and cancer.^8–10^ However, the risks associated with the IMIDs themselves are less understood. Two large cohort studies compared adult patients with and without IMIDs and found that mortality from COVID-19 was higher among those with IMIDs.^9,11^ Similarly, some studies have indicated an association between the use of IMMs and an increased risk of severe COVID-19 outcomes,^12^ although others have not.^7,13^

Previous studies analysing IMIDS, IMMs, and comorbidities in cohorts of patients with COVID-19^9,11,14–16^ did not report SARS-CoV-2 omicron (B.1.1.529, BA.1.1, BA.2, BA.2.12.1, BA5) variants or COVID-19 vaccination status, and were conducted in cohorts of fewer than 1000 patients.^17^ A 2023 study that used data from the National COVID Cohort Collaborative, which spans the period in the pandemic both before and after the emergence of the omicron variant, showed that patients with a previous IMID or previous exposure to IMMs had an increased risk of life-threatening outcomes from COVID-19.^18^ However, the study design grouped different IMIDs together, making it difficult to decipher the role of individual IMIDs in the outcomes of patients with COVID-19.

Using multivariable models across a large US population, we aimed to analyse severe COVID-19 outcomes, including hospitalisation, mechanical ventilation, and death, among patients with IMIDs and to investigate the risk associated with individual IMIDs, classes of IMMs, chronic comorbidities, and COVID-19 vaccination status. The study period was dichotomised so that we could compare the periods before and after the emergence of the omicron variant during the COVID-19 pandemic.

## Methods

### Study design and participants

In this retrospective cohort study, clinical data were derived from electronic health records (EHRs) from Providence St Joseph Health (PSJH), an integrated health-care system that serves patients in 51 hospitals and 1085 clinics across seven US states: Alaska, California, Montana, Oregon, New Mexico, Texas, and Washington. Two timeframes were analysed: March 1, 2020–Dec 25, 2021 (the pre-omicron period), when the wild-type (B) alpha (B.1.1.7), beta (B.1.351), and delta (P.1) variants were predominant; and Dec 26, 2021–Aug 30, 2022 (the omicron-predominant period).

Patients with IMIDs (no age restriction) were identified on the basis of their medical history, and all unmatched controls of patients who tested positive for COVID-19 and did not have an IMID were selected from the same database. Data were observed for patients with a valid nucleic acid amplification test for SARS-CoV-2. To ensure information on IMIDs, medications, and comorbidities was known before a patient’s first COVID-19 infection, patients were included if they had at least one encounter at PSJH at least 2 weeks before their first COVID-19 test.

This observational study followed STROBE guidelines (appendix pp 23–27). All procedures were reviewed and approved by the Institutional Review Board at PSJH through expedited review (STUDY2021000592). Patient consent was waived because disclosure of protected health information for the study was determined to involve no more than a minimal risk to the privacy of individuals.

### Procedures

**Figure 1:**
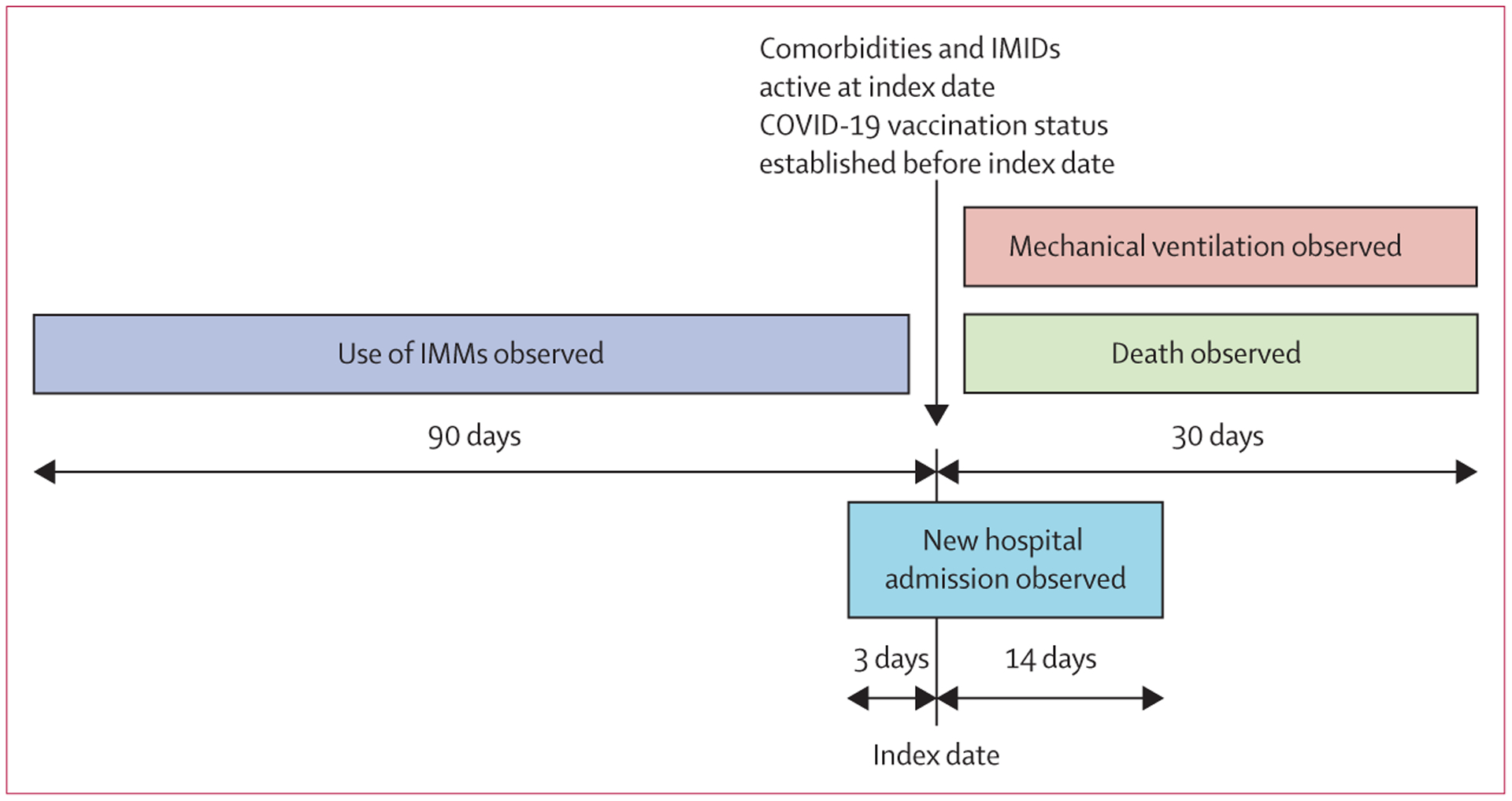
Study timeline for observation of characteristics and outcomes For patients with COVID-19, the index date was date of first positive test; for patients without COVID-19, the index date was date of first negative test. IMID=immune-mediated inflammatory disease. IMM=immunomodulatory medication.

The index date was considered to be the date of a valid COVID-19 test (infection date or first negative test). For patients with COVID-19, the index date was set to the date of their first positive test; for those without COVID-19, the index date was set to the date of their first negative test. Patients with severe COVID-19 outcomes (ie, hospitalisation, mechanical ventilation, or death) were identified if they were hospitalised (new admission) within the window of 3 days before 14 days of the index date; or received mechanical ventilation or died within 30 days of the index date (figure 1).

Patient use of IMMs was observed for 3 months leading up to the index date to include medications that were administered periodically and had a multiple-month effect on the immune system (figure 1). IMMs were identified by RxNorm medication order codes (appendix pp 20–21). To be able to establish the effect of the use of IMMs at the time of SARS-CoV-2 infection, we selected a subset of patients who had at least one encounter with PSJH before the index date.

Comorbidities and IMIDs were identified by diagnosis codes by use of SNOMED-CT (version obtained on June 27, 2022; appendix pp 18–20). The active status of patient comorbidities and IMIDs was decided based on the index date. COVID-19 vaccination status was decided before the index date. For patients with a COVID-19 vaccine from either Moderna or Pfizer, we counted two administered doses as fully vaccinated and more than two doses as boosted. For patients with a COVID-19 vaccine from Janssen, we counted one administered dose as fully vaccinated and more than one dose as boosted. All vaccination information was obtained from state records and limited to the seven states in our study. If patients only came for a COVID-19 test during the pandemic they would not necessarily have a history taken and any comorbidities, vaccination status, and previous use of IMMs would be recorded as unknown.

Primary outcomes were the combined endpoint of hospitalisation, mechanical ventilation, and death; the combined endpoint of mechanical ventilation and death; and death among patients with COVID-19. Hospitalisation is defined within the window of 3 days before the index date to 14 days after the index date. Mechanical ventilation and death are defined as the window within 30 days after the index date. We assessed differences in the rates of outcomes using Fisher’s exact test.

### Statistical analysis

To evaluate which variables were most predictive of severe COVID-19 outcomes, we trained supervised machine learning models on 62 features of two cohorts of patients testing positive for SARS-CoV-2. Variables were patient demographics, COVID-19 vaccination and booster status, active comorbidities, diagnoses of IMIDs, and use of IMMs.

Continuous variables, age, and BMI were normalised by applying min–max transformation. Missing data were addressed by use of the median value to impute missing BMI values and assuming the absence of active comorbidities, IMIDs, use of IMMs, and vaccination when such data were not reported as active in structured EHR data.

Two alternate analyses were conducted to analyse anti-SARS-CoV-2 neutralising monoclonal antibodies: one with an additional binary variable for the administration of antibodies within 10 days of COVID-19 test results, and one excluding patients who received these antibodies. An additional analysis was also conducted with nirmatrelvir–ritonavir.

We selected two machine learning methods to assess the relative importance of IMIDs, IMMs, and comorbidities as risk factors for classifying severe COVID-19 outcomes: traditional logistic regression (LR), for ease of interpretability; and extreme gradient boosted decision tree (XGB), which is an efficient implementation of the regularised gradient boosted decision tree model that can learn non-linear relationships from high-dimensional datasets and achieve good performance without cost-prohibitive computing requirements. Three additional modelling approaches were assessed to test the assumption that XGB would have the highest performance: adaptive boosting, the k-nearest neighbour algorithm, and support vector machine (appendix p 2).

Models were generated with the Python package Scikit-Learn (version 1.1.1) and XGB (version 1.6.1). Factors with fewer than ten observations were excluded from the LR model and XGB hyperparameters were tuned with the sklearn.model_selection.RandomizedSearchCV function (appendix p 11) using ten-fold cross-validation. Models were trained on 90% of the data with an over-sampling method for the LR model and an over-weight method (controlled by the scale_pos_weight parameter; appendix p 11) on minority classes to address class imbalance in training data, with 10% of the data held out for independent performance testing of the final models. Performance was evaluated on the test set for the area under the receiver operating characteristic curve, calculated with the function metrics.roc_auc_score from the Python package Scikit-Learn (version 1.1.1), with the parameter average defined as weighted. Models were also evaluated by plotting the log-transformed adjusted odds ratio (OR) for each feature.

Feature importance and Shapley Additive Explanations (SHAP) were applied to understand each variable’s marginal contribution by use of the Python libraries Scikit-Learn and its influence on model prediction by use of SHAP (version 0.37.0).^19^ Variable independence was assessed with the variance inflation factor method. A value equal to one indicates no correlation; a value greater than 5·00 is considered to be an indicator that a feature is highly correlated with other features and needs additional feature engineering. The Benjamini–Yekutieli multiple hypothesis correction was applied to p values by use of the Python statsmodel package (version 0.12.2) and both uncorrected and corrected values were reported. A p value of less than 0·05 was considered to be statistically significant in this study after the hypothesis correction was applied.

Data processing and machine learning models were conducted on Microsoft Azure with Databricks 9.1 LTS, which includes Apache Spark 3.1.2. All analysis codes were implemented and performed in Python (version 3.8.10), except for the figures, which were plotted in R (version 4.1.1).

### Role of the funding source

The funders of the study had no role in study design, data collection, data analysis, data interpretation, or writing of the report.

## Results

Of 2 167 656 patients tested for SARS-CoV-2, 290 855 (13·4%) had confirmed COVID-19: 15 397 (5·3%) patients with IMIDs and 275 458 (94·7%) without IMIDs. The majority of people testing positive for COVID-19 in both the pre-omicron period (March 1, 2020–Dec 25, 2021; 110 217 [64·8%] individuals) and the omicron-predominant period (Dec 26, 2021–Aug 30, 2022; 64 864 [53·7%] individuals) were not fully vaccinated (table 1). In the omicron-predominant period, both patients tested for COVID-19 and those with a positive test result had higher rates of comorbidities and a higher rate of vaccination than did those in the pre-omicron period (table 1).

**Figure 2:**
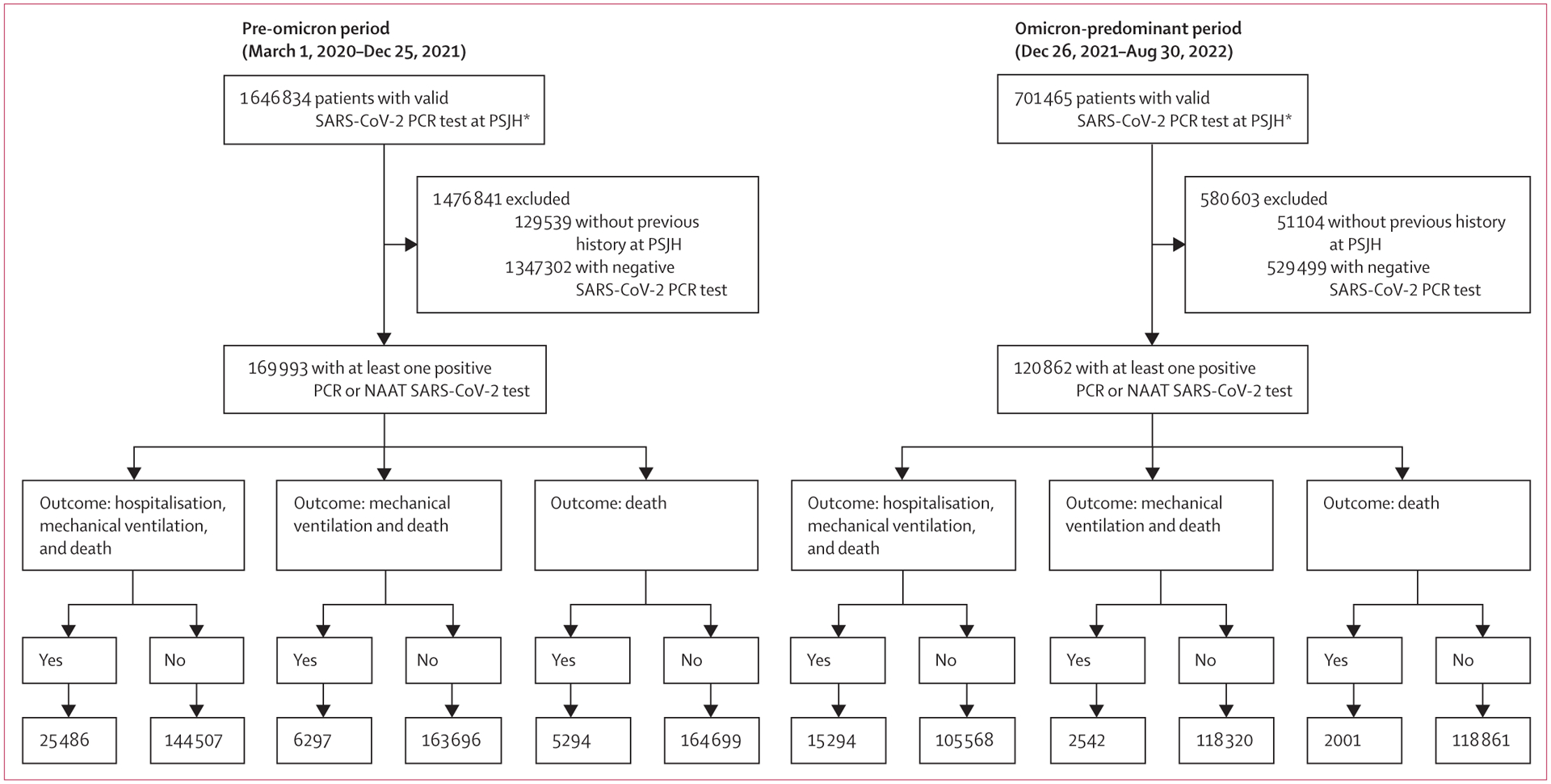
Cohort flow diagram over the pre-omicron and omicron-predominant periods NAAT=nucleic acid amplification test. PSJH=Providence St Joseph Health. *Total patient number including patients without previous history at PSJH.

In the pre-omicron period, 169 993 (11·2%) of 1 517 295 people who were tested for COVID-19 tested positive, of whom 23 330 (13·7%) were hospitalised, 1072 (0·6%) received mechanical ventilation, and 5294 (3·1%) died (figure 2; table 2). Among patients with IMIDs who underwent testing, 8070 (9·7%) of 83 497 patients tested positive for COVID-19. Compared with controls, patients with IMIDs and COVID-19 had higher rates of hospitalisation (1176 [14·6%] *vs* 22 154 [13·7%]; p=0·024), and mortality (314 [3·9%] *vs* 4980 [3·1%]; p<0·0001). In the omicron-predominant period, the overall rate of individuals testing positive for COVID-19 increased to 18·6% (120 862 of 650 361 patients); however, rates of hospitalisation (14 504 [12·0%]), mechanical ventilation (567 [0·5%]), and death (2001 [1·7%]) decreased (table 2). In this period, compared with controls, patients with IMIDs and COVID-19 (7327 [17·3%] of 42 249) had higher rates of hospitalisation (1082 [14·8%] *vs* 13 422 [11·8%]; p<0·0001) and death (187 [2·6%] *vs* 1814 [1·6%]; p<0·0001).

**Figure 3:**
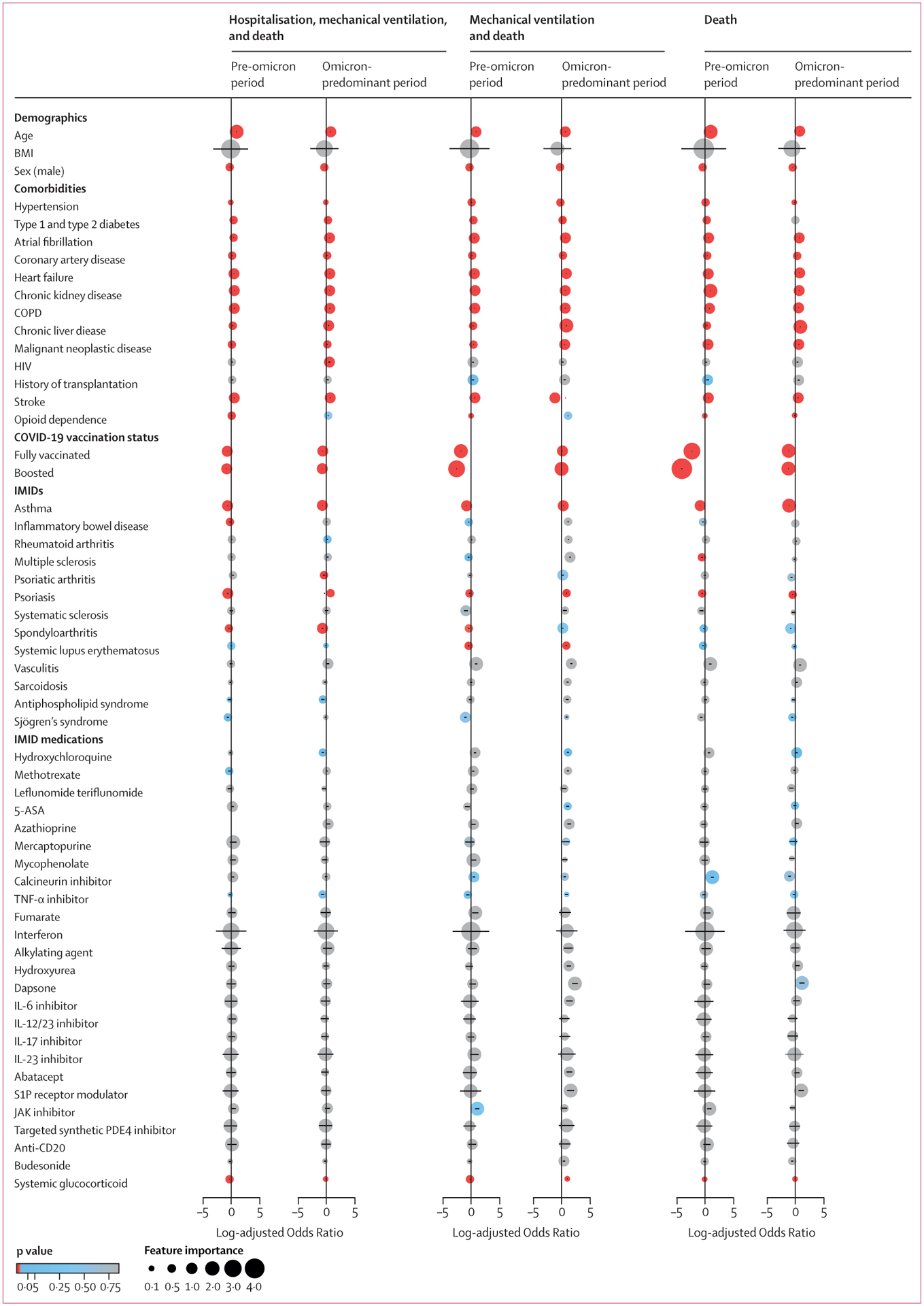
Selected factors for hospitalisation, mechanical ventilation, and death in patients with COVID-19 between pre-omicron and omicron-predominant periods The pre-omicron period was between March 1, 2020, and Dec 25, 2021; the omicron-predominant period was between Dec 26, 2021, and Aug 30, 2022. The colour of each circle represents its corresponding p value, calculated with raw data. The position and size of each circle represents log-adjusted ORs and feature importance from over-sampling. Log-adjusted ORs were calculated with multivariable logistic regression. Error bars within circles represent 95% CIs. Factors with fewer than ten observations were excluded. COPD=chronic obstructive pulmonary disorder. IMID=immune-mediated inflammatory disease.

Several results were significant across both time periods for all three severe COVID-19 outcomes (figure 3; appendix pp 10–18). Age was a risk factor for worse outcomes (adjusted OR from 2·1 [95% CI 2·0–2·1]; p<0·0001 to 3·0 [2·9–3·0]; p<0·0001), whereas COVID-19 vaccination (from 0·082 [0·080–0·085]; p<0·0001 to 0·52 [0·50–0·53]; p<0·0001) and booster vaccination (from 2·1 [2·0–2·2]; p<0·0001 to 3·0 [2·9–3·0]; p<0·0001) status were associated with better outcomes. Seven comorbidities were risk factors: atrial fibrillation (adjusted OR from 1·6 [1·5–1·6]; p<0·0001 to 2·3 [2·2–2·3]; p<0·0001), coronary artery disease from 1·2 [1·1–1·2]; p<0·0001 to 1·5 [1·5–1·6]; p<0·0001), heart failure (from 1·7 [1·6–1·7]; p<0·0001 to 2·6 [2·5–2·7]; p<0·0001), chronic kidney disease (from 1·8 [1·7–1·8]; p<0·0001 to 2·8 [2·7–2·9]; p<0·0001), COPD (from 1·8 [1·7–1·8]; p<0·0001 to 2·0 [2·0–2·1]; p<0·0001), chronic liver disease (from 1·3 [1·3–1·4]; p<0·0001 to 2·8 [2·6–3·0]; p<0·0001), and malignant neoplastic disease (from 1·1 [1·1–1·2]; p<0·0001 to 2·1 [2·0–2·1]; p<0·0001). Two IMIDs, asthma (adjusted OR from 0·33 [0·32–0·34] to 0·49 [0·48–0·51]; p<0·0001) and psoriasis (from 0·52 [0·48–0·56] to 0·80 [0·74–0·87]; p<0·0001), were associated with a reduced risk of severe outcomes. IMID diagnoses did not appear to be significant risk factors themselves, but results were limited by small sample size, and vasculitis had high feature importance in LR. IMMs did not appear to be significant, but less frequently used IMMs were limited by sample size.

**Figure 4:**
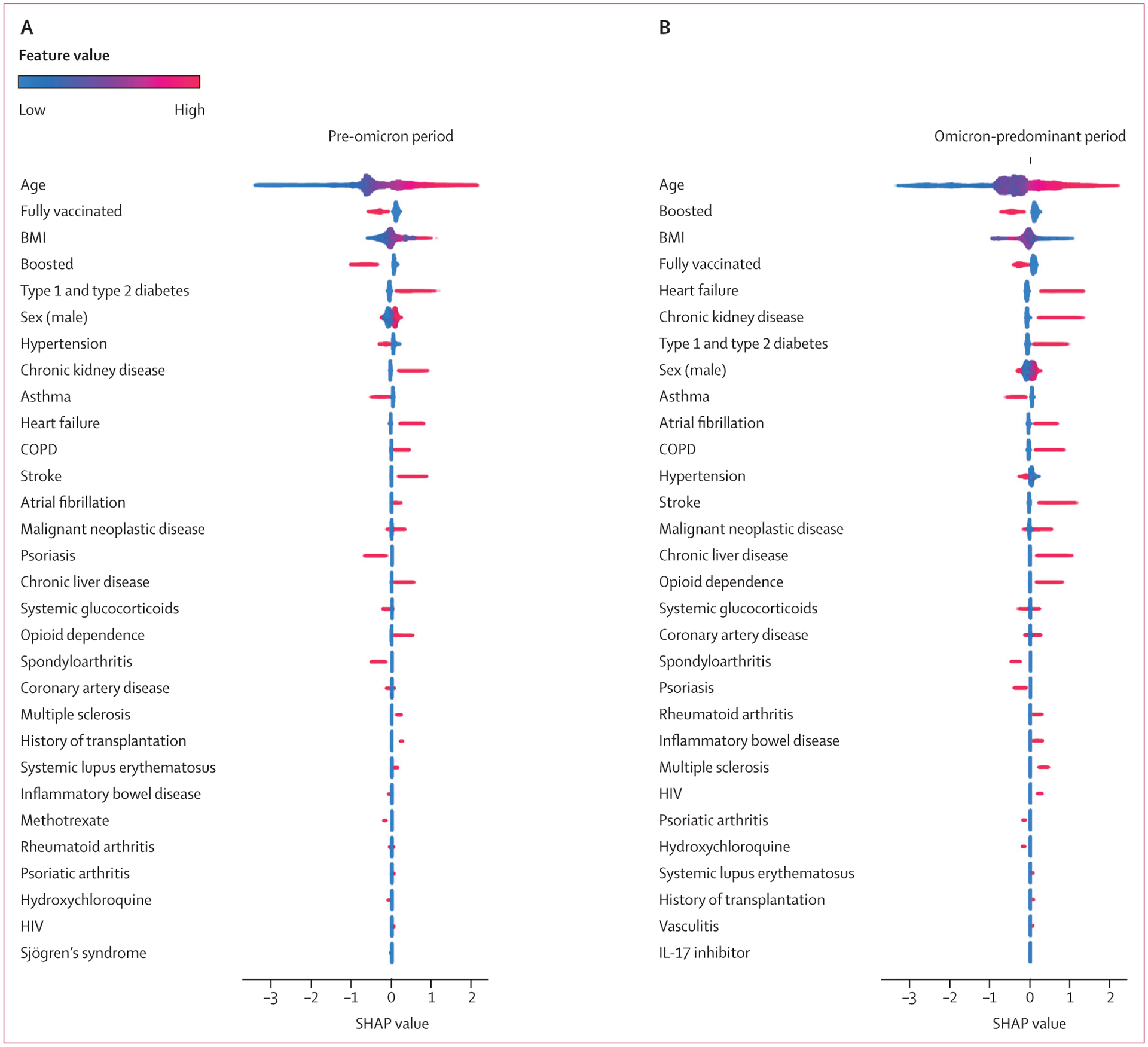
SHAP summary plot for top 30 features of all-age model of hospitalisation, mechanical ventilation, and death among patients with COVID-19 Feature importance provided for the pre-omicron period between March 1, 2020 and Dec 25, 2021 (A) and the omicron-predominant period between Dec 26, 2021 and Aug 30, 2022 (B). Gradient boosting decision tree feature importance and influence of higher and lower values of the risk factors on the all-age group population outcome. SHAP values of less than 0 are associated with a reduced risk of hospitalisation, mechanical ventilation, or death; SHAP values of more than 0 are associated with an increased risk. Red dots represent patients with higher values for a variable; blue dots represent patients with lower values. Nominal classes are binary (0 or 1). For sex, male is 1 (red). COPD=chronic obstructive pulmonary disorder. SHAP=Shapley Additive Explanations.

**Figure 5:**
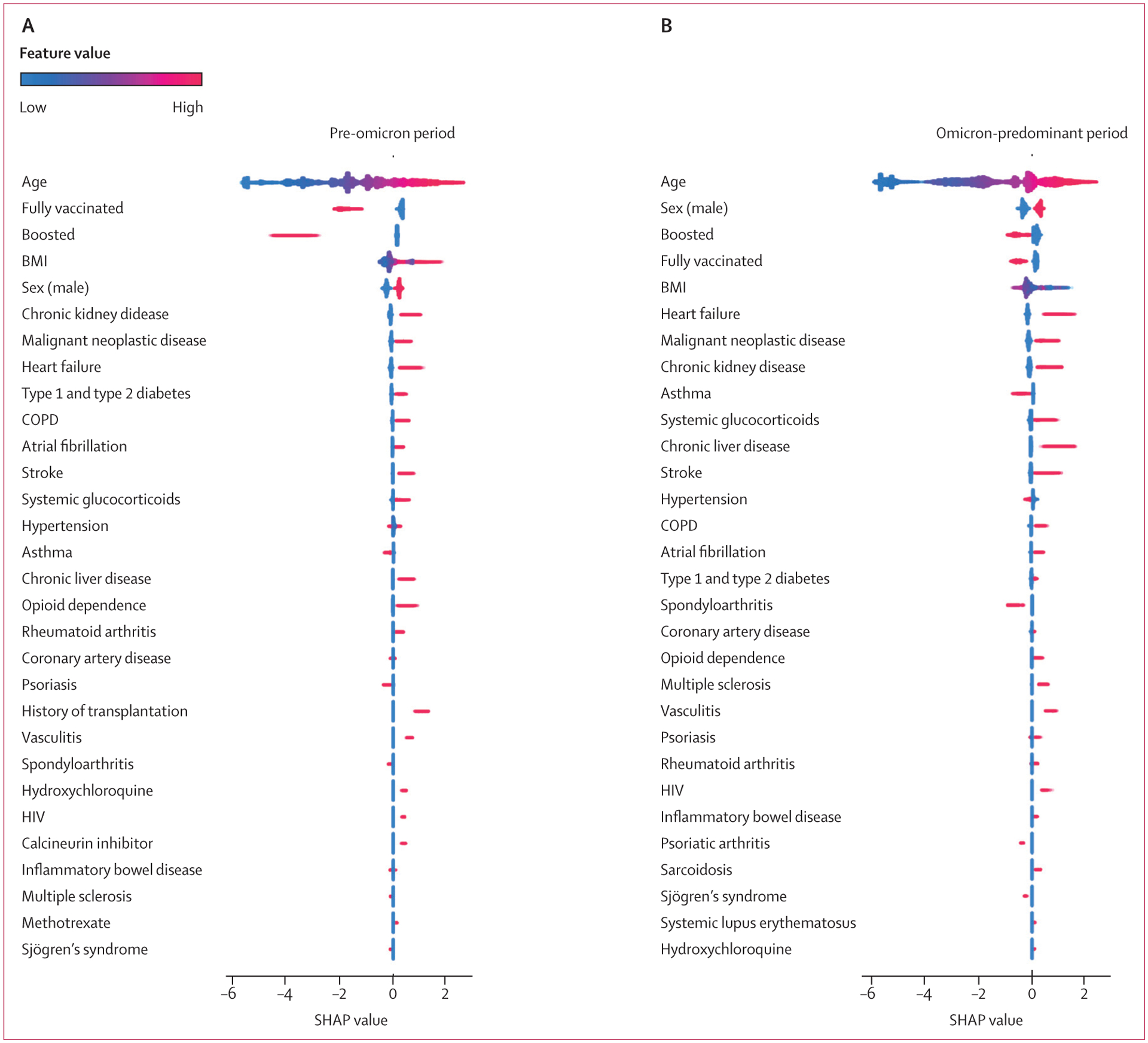
SHAP summary plot for top 30 features of all-age model of death among patients with COVID-19 Feature importance provided for the pre-omicron period between March 1, 2020, and Dec 25, 2021 (A) and the omicron-predominant period between Dec 26, 2021 and Aug 8, 2022 (B). Gradient boosting decision tree feature importance and influence of higher and lower values of the risk factors on the all-age group population outcome. SHAP values of less than 0 are associated with a reduced risk of hospitalisation, mechanical ventilation, or death; SHAP values of more than 0 are associated with an increased risk. Red dots represent patients with higher values for a variable; blue dots represent patients with lower values. Nominal classes are binary (0 or 1). For sex, male is 1 (red). COPD=chronic obstructive pulmonary disorder. SHAP=Shapley Additive Explanations.

XGB area under the receiver operating characteristic curve for the classification of all health outcomes in both time periods (range 0·77–0·92) outperformed LR (0·70–0·84). On average, XGB had 7·5% better classification performance on the hold out test set than LR, across all three outcomes and both time periods (appendix p 2). The majority of results, age, chronic cormorbidities, and COVID-19 vaccination and booster status from the SHAP analysis on the XGB model showed similar associations and relative feature importance seen in LR (figures 4, 5; appendix pp 4–6). As with the LR model, results classified improved outcomes for patients with asthma, spondyloarthritis, and psoriasis (with the exception that psoriasis classified worse outcomes for death in the omicron-predominant time period; figures 4, 5; appendix pp 4–6). Furthermore, XGB and SHAP showed that opioid dependence was predictive of all severe outcomes in both time periods, and rheumatoid arthritis, multiple sclerosis, and vasculitis were predictive of all three severe outcomes in the omicron-predominant period (figures 4, 5; appendix pp 4–6). Long-term use of systemic glucocorticoids showed mixed results for hospitalisation, but was predictive of mechanical ventilation in the omicron-predominant period and of death in both time periods (figures 4, 5; appendix pp 4–6). The results of the analyses on anti-SARS-CoV-2 neutralising monoclonal antibodies are provided in the appendix (pp 8–10). The results of the analysis on nirmatrelvir–ritonavir are provided in the appendix (pp 10–11).

## Discussion

In this large retrospective cohort study, we showed that patients with IMIDs had reduced rates of COVID-19 but increased rates of severe COVID-19 outcomes during both the pre-omicron and omicron-predominant periods of the pandemic. Having a specific IMID diagnosis was less predictive of severe COVID-19 outcomes than was age, whereas vaccination and booster status were protective. Rheumatoid arthritis, vasculitis, and multiple sclerosis had some predictive value for all three severe outcomes (hospitalisation, mechanical ventilation, and death) during the omicron-predominant period.

Asthma was associated with a reduced risk of all three outcomes, relative to the population overall, supporting previous research suggesting it might have some protective effect.^5^ Interestingly, psoriasis also showed a reduced risk of all three outcomes in both time periods, and spondyloarthritis was associated with a reduced risk in the pre-omicron period. These findings are similar to previous findings of spondyloarthritis reported by Raiker and colleagues.^20^ By contrast, Rosenbaum and colleagues^21^ reported a small increased risk of developing COVID-19 in patients with spondyloarthritis in the pre-omicron period, although this risk was not consistently shown. Although this observation might reflect unmeasured variables, such as behaviours taken to avoid risk of infection, the results merit further investigation into whether psoriasis or spondyloarthritis are associated with factors that might reduce susceptibility to SARS-CoV-2 or COVID-19 severity, such as some alleles of HLA-B15.^22–24^

The most important factors for the combined endpoint of hospitalisation, mechanical ventilation, or death were age, diabetes, chronic kidney disease, COPD, stroke, atrial fibrillation, liver disease, and opioid dependence. The strongest risk factors for mortality were age, pre-existent heart failure, chronic kidney disease, atrial fibrillation, COPD, stroke, and liver disease. BMI above 30 kg/m^2^ has been associated with worse outcomes;^8^ however, SHAP results on gradient boosting show mixed results. This observation could suggest that risks can come from having underweight or obesity, or might reflect that the predictive importance of BMI differs between younger (aged <50 years) and older (aged ≥50 years) patients.^25^ Our results support previously reported studies on the risks of comorbidities and benefits of COVID-19 vaccination in the pre-omicron period.^10,26,27^ Additionally, we show that these associations continued into the omicron-predominant period, and that booster vaccination is also predictive of improved outcomes.

A UK nationwide cohort study on the OpenSAFELY platform showed that patients with autoimmune diseases, such as rheumatoid arthritis, systemic lupus erythematosus, and psoriasis, who tested positive for COVID-19 in the pre-omicron period had an increased risk of death, compared with those without autoimmune disease.^28^ In some analyses, we observed an increased risk of severe COVID-19 outcomes in patients with rheumatoid arthritis; however, we found improved outcomes for patients with psoriasis in both pandemic periods. Of note, Piaserico and colleagues^29^ evaluated the quality of previous studies related to outcomes of COVID-19 in patients with psoriatic arthritis and psoriasis treated with IMMs, and found a risk of bias.

Overall, our results support previously noted associations between specific IMIDs and outcomes from the pre-omicron period of the pandemic,^6,27,30^ provide new results for the omicron-predominant period, and suggest priorities for further investigation into the increased risks observed in patients with rheumatoid arthritis and multiple sclerosis, and the reduced risks (and potential protective benefits) associated with psoriasis and spondyloarthritis. Certain IMMs were previously reported to be associated with worse outcomes.^26,27,31,32^ Long-term use of glucocorticoids only showed an adjusted OR of 1·02 (95% CI 0*·*98–1·06) for death during the omicron-predominant period in results from the LR model, but showed predictive value for an increased risk of mortality in results from the XGB model for both pandemic periods. This finding supports previous reports that long-term use of systemic glucocorticoids could be implicated in adverse outcomes for some patients with IMIDs in the pre-omicron period.^10,14,32,33^ However, further research is needed on IMID severity at the time of COVID-19, given that disease flares might be a confounding factor affecting both steroid treatment and COVID-19 outcomes. Unlike data from prospective IMID studies, real world EHR data rarely includes structured information on IMID severity. Severity is usually mentioned only in free text notes, in heterogeneous ways, and often with minimal detail. Long-term use of other IMMs was not clearly predictive of worse outcomes. However, the analysis of less frequently used IMMs was limited by the sample size and requires further study. For example, the number of patients on anti-CD20 who tested positive for COVID-19 was low (only 12 patients in the pre-omicron period and 16 in the omicron-predominant period).

The results of our study suggest that, for patients with IMIDs, age, comorbidities, and not being fully vaccinated are more important predictive factors of severe COVID-19 outcomes than long-term use of IMMs. However, care should be taken in interpreting population-wide results. Correcting for a false discovery rate with multiple hypothesis testing reduces the risk of reporting a spurious signal, but increases the risk of overlooking factors that might be important for a subset of patients. For example, other large population studies have noted an increased risk of severe COVID-19 outcomes with long-term used of rituximab, an anti-CD20 IMM.^27,31,34^

The strengths of our study include a large population sample size, a multivariable analysis with a focus on immune-related risk factors, and the application of two complementary modelling approaches. We also investigated the full pandemic time period, comparing results from the early phase with those from the omicron-predominant period to provide insight into whether risk factors have changed since the emergence of the omicron variant.

Limitations of this study include a scarcity of information on: IMID disease severity and activity; the severity of non-IMID comorbidities; patients with COVID-19 who were not tested at PSJH; several aspects of SARS-CoV-2 immunity, including infection-induced immunity, diminishing effects from immunisation over time, and differences in immunity to different variants; sequencing data on actual variants; the intensity or dosage of IMMs; and the rate of IMID underdiagnoses or billing code errors. There was also no distinction between medication dosing and differences in total timing and duration of medications during the 3 months leading up to SARS-CoV-2 infection, which is of particular importance for deciphering the role of systemic glucocorticoids in COVID-19 outcomes. Differences in standard of care treatment for COVID-19 were partially accounted for by having two time periods; however, the analysis did not include COVID-19 treatment medications at the per-patient level (other than the additional analysis for anti-SARS-CoV-2 neutralising monoclonal antibodies), and patients with IMIDs might have been treated differently due to clinician concern for an increased risk of severe COVID-19 outcomes. Additionally, particularly in the omicron-predominant period, at-home rapid tests became more readily available, leading to a lower reported rate of severe outcomes in individuals testing positive for COVID-19 than the rates reported in this study.

Although variance inflation factors were considered sufficiently low (<5·00), age had a value of 3·58 in the omicron-predominant period, suggesting opportunities for future research into age-stratified models.^29^ Furthermore, we might have missed other potentially confounding factors, such as behavioural choices for patients with immune-related conditions and medications, other socioeconomic exposures, and delays in access to care. In addition, some sample bias might have been introduced when only selecting patients who had previously received care at PSJH. Although this inclusion criterion was needed to establish the long-term use of IMMs, it might have excluded patients who had barriers to accessing care and only sought COVID-19 treatment if they were extremely ill.

In this Article, we analysed 2 167 656 patients tested for COVID-19 from a large US health-care system database. For patients with a positive COVID-19 test result, we developed predictive models for severe COVID-19 outcomes (hospitalisation, mechanical ventilation, and death) on 15 397 patients with IMIDs and 275 458 unmatched controls. We examined two separate time periods of the pandemic: the pre-omicron period and the omicron-predominant period. Patients with IMIDs had higher rates of hospitalisation and mortality than did patients with COVID-19 without IMIDs. Using multivariable LR and XGB models, we showed that age and chronic comorbidities were risk factors for severe COVID-19 outcomes, whereas vaccination and boosters were associated with a reduced risk. However, apart from rheumatoid arthritis and multiple sclerosis, the specific IMIDs themselves did not show an association with worse outcomes. Interestingly, spondyloarthritis and psoriasis were associated with improved outcomes, which suggests that, like asthma,^8^ these IMIDs might point to new insights on protective mechanisms against COVID-19.

IMMs were not significantly associated with worse outcomes. Certain classes of IMMs showed some predictive value; however, due to an absence of significance after correcting for a false discovery rate and inconsistent results across analytical methods and time periods, these observations need further investigation. Furthermore, some IMMs might have clinically significant benefits or harms for a subset of patients with IMIDs. In general, care should be taken when interpreting the results of the study. Given unmeasured potential confounders and unmatched controls, risk factors should not necessarily be deemed as causal. However, the results from these more comprehensive multivariate models can help to inform clinical, policy, and research decisions for patients with IMIDs and COVID-19. Overall, there is a need to take age and comorbidities into consideration when developing COVID-19 guidelines for patients with IMIDs. Further research is needed for specific IMIDs (including IMID severity at the time of SARS-CoV-2 infection) and IMMs (considering dosage and timing before a patient’s first COVID-19 infection).

## Supplementary Material

1

## Figures and Tables

**Table 1:** Demographic and clinical characteristics of patients with IMIDs at time of first confirmed COVID-19 diagnosis

	Pre-omicron period (March 1, 2020–Dec 25, 2021)		Omicron-predominant period (Dec 26, 2021–Aug 30, 2022)	
	Tested for COVID-19 (n=1 517 295)*	Positive for COVID-19 (n=169 993)	Tested for COVID-19 (n=650 361)*	Positive for COVID-19 (n=120 862)
**Age group, years**				
0–17	138 738 (9·1%)	15 658 (9·2%)	67 279 (10·3%)	14 962 (12·4%)
18–49	563 633 (37·1%)	77 054 (45·3%)	217 755 (33·5%)	50 222 (41·6%)
50–74	589 961 (38·9%)	58 418 (34·4%)	247 139 (38·0%)	38 521 (31·9%)
≥75	224 963 (14·8%)	18 863 (11·1%)	118 188 (18·2%)	17 157 (14·2%)
Age, years	52·0 (32·0–68·0)	46·0 (29·0–63·0)	54·0 (33·0–70·0)	46·0 (28·0–66·0)
**Weight status**				
BMI, kg/m^2^	28·5 (24·2–34·2)	29·1 (25·1–35·3)	28·5 (24·1–34·5)	28·5 (24·1–34·8)
Unknown	66 718 (4·4%)	9384 (5·5%)	16 234 (2·5%)	4075 (3·4%)
**Sex**				
Female	868 765 (57·3%)	92 943 (54·7%)	374 472 (57·6%)	69 040 (57·1%)
Male	648 319 (42·7%)	77 039 (45·3%)	275 769 (42·4%)	51 808 (42·9%)
Unknown	211 (0·0%)	11 (0·0%)	120 (0·0%)	14 (0·0%)
**Race**				
White	1 112 468 (73·3%)	113 111 (66·5%)	469 672 (72·2%)	81 501 (67·4%)
Multiple or other	172 068 (11·3%)	31 264 (18·4%)	80 617 (12·4%)	19 135 (15·8%)
Asian	76 146 (5·0%)	5927 (3·5%)	32 642 (5·0%)	5922 (4·9%)
Unknown	48 418 (3·2%)	8704 (5·1%)	28 213 (4·3%)	5887 (4·9%)
Black	53 631 (3·5%)	6463 (3·8%)	25 148 (3·9%)	5536 (4·6%)
American Indian and Alaska Native	20 036 (1·3%)	2680 (1·6%)	9168 (1·4%)	1770 (1·5%)
Native Hawaiian and other Pacific Islander	10 621 (0·7%)	1844 (1·1%)	4901 (0·8%)	1111 (0·9%)
**COVID-19 vaccination status**				
Fully vaccinated without booster	426 793 (28·1%)	44 637 (26·3%)	147 029 (22·6%)	30 982 (25·6%)
Fully vaccinated with booster	293 413 (19·3%)	15 139 (8·9%)	204 783 (31·5%)	25 016 (20·7%)
Not fully vaccinated	797 089 (52·5%)	110 217 (64·8%)	298 549 (45·9%)	64 864 (53·7%)
**COVID-19 vaccination type**				
Pfizer-BioNTech (BNT162b2)	351 541 (23·2%)	30 303 (17·8%)	169 485 (26·1%)	28 432 (23·5%)
Moderna (mRNA-1273)	309 377 (20·4%)	22 315 (13·1%)	154 929 (23·8%)	22 415 (18·5%)
Janssen (JNJ-78436735)	59 288 (3·9%)	7158 (4·2%)	27 398 (4·2%)	5151 (4·3%)
**COVID-19 treatment after first positive COVID-19 date**				
Anti-SARS-CoV-2 monoclonal antibody (bamlanivimab, bebtelovimab, casirivimab, cilgavimab, etesevimab, imdevimab, sotrovimab, or tixagevimab)	703 (0·0%)	699 (0·4%)	2937 (0·5%)	2822 (2·3%)
Nirmatrelvir-ritonavir	NA	NA	NA	132 (0·1%)
**Comorbidities**				
Hypertension	314 231 (20·7%)	31 454 (18·5%)	162 657 (25·0%)	26 284 (21·7%)
Type 1 and type 2 diabetes	142 312 (9·4%)	17 822 (10·5%)	77 158 (11·9%)	13 697 (11·3%)
Chronic kidney disease	92 178 (6·1%)	9712 (5·7%)	55 400 (8·5%)	9309 (7·7%)
Coronary artery disease	95 874 (6·3%)	8328 (4·9%)	53 394 (8·2%)	7913 (6·5%)
Atrial fibrillation	91 596 (6·0%)	7710 (4·5%)	53 994 (8·3%)	7530 (6·2%)
Heart failure	77 398 (5·1%)	7581 (4·5%)	48 872 (7·5%)	7601 (6·3%)
Stroke	34 944 (2·3%)	3222 (1·9%)	21 924 (3·4%)	3405 (2·8%)
Chronic obstructive pulmonary disease	70 258 (4·6%)	6257 (3·7%)	40 088 (6·2%)	6245 (5·2%)
Chronic liver disease	22 972 (1·5%)	2158 (1·3%)	12 927 (2·0%)	2057 (1·7%)
Active malignant neoplasm	175 207 (11·5%)	14 916 (8·8%)	92 365 (14·2%)	13 801 (11·4%)
HIV	2957 (0·2%)	276 (0·2%)	1496 (0·2%)	262 (0·2%)
History of transplantation	1534 (0·1%)	216 (0·1%)	954 (0·1%)	210 (0·2%)
Opioid dependence	22 996 (1·5%)	2247 (1·3%)	12 987 (2·0%)	2107 (1·7%)
**IMIDs before first positive COVID-19 date** †				
Total*	1 517 295 (100·0%)	169 993 (100·0%)	650 361 (100·0%)	120 862 (100·0%)
Asthma	141 513 (9·3%)	16 049 (9·4%)	69 956 (10·8%)	13 816 (11·4%)
Psoriasis	20 797 (1·4%)	2100 (1·2%)	10 082 (1·6%)	1824 (1·5%)
Rheumatoid arthritis	18 539 (1·2%)	1941 (1·1%)	9931 (1·5%)	1786 (1·5%)
Inflammatory bowel disease	14 979 (1·0%)	1242 (0·7%)	7148 (1·1%)	1114 (0·9%)
Spondyloarthritis	13 182 (0·9%)	1318 (0·8%)	7215 (1·1%)	1241 (1·0%)
Systemic lupus erythematosus	6091 (0·4%)	661 (0·4%)	3272 (0·5%)	648 (0·5%)
Multiple sclerosis	6211 (0·4%)	612 (0·4%)	3134 (0·5%)	559 (0·5%)
Psoriatic arthritis	4941 (0·3%)	486 (0·3%)	2655 (0·4%)	450 (0·4%)
Sjögren’s syndrome	3849 (0·3%)	314 (0·2%)	2041 (0·3%)	321 (0·3%)
Sarcoidosis	2890 (0·2%)	260 (0·2%)	1414 (0·2%)	228 (0·2%)
Antiphospholipid syndrome	1497 (0·1%)	121 (0·1%)	802 (0·1%)	143 (0·1%)
Systemic sclerosis	1368 (0·1%)	110 (0·1%)	694 (0·1%)	97 (0·1%)
Vasculitis	1569 (0·1%)	169 (0·1%)	884 (0·1%)	200 (0·2%)
**IMMs before first positive COVID-19 date**				
Hydroxychloroquine	2285 (0·2%)	275 (0·2%)	1678 (0·3%)	335 (0·3%)
Methotrexate	2637 (0·2%)	293 (0·2%)	1623 (0·2%)	325 (0·3%)
Leflunomide teriflunomide	624 (0·0%)	75 (0·0%)	445 (0·1%)	72 (0·1%)
5-ASA (balsalazide, sulfasalazine, or mesalamine)	1596 (0·1%)	131 (0·1%)	917 (0·1%)	152 (0·1%)
Azathioprine	603 (0·0%)	59 (0·0%)	335 (0·1%)	74 (0·1%)
Mercaptopurine	102 (0·0%)	16 (0·0%)	75 (0·0%)	19 (0·0%)
Mycophenolate	198 (0·0%)	42 (0·0%)	156 (0·0%)	48 (0·0%)
Calcineurin inhibitor (ciclosporin, sirolimus, or tacrolimus)	1373 (0·1%)	180 (0·1%)	945 (0·1%)	227 (0·2%)
TNF-α inhibitor (adalimumab, certolizumab pegol, etanercept, golimumab, or infliximab)	1628 (0·1%)	191 (0·1%)	944 (0·1%)	193 (0·2%)
Fumarate (dimethyl, diroximel, or monomethyl)	148 (0·0%)	17 (0·0%)	76 (0·0%)	16 (0·1%)
Interferon (interferon beta-1a or interferon beta-1b)	53 (0·0%)	2 (0·0%)	25 (0·0%)	4 (0·0%)
Alkylating agent (chlorambucil)	54 (0·0%)	5 (0·0%)	38 (0·0%)	8 (0·0%)
Hydroxyurea	288 (0·0%)	27 (0·0%)	211 (0·0%)	40 (0·0%)
Dapsone	133 (0·0%)	19 (0·0%)	77 (0·0%)	20 (0·0%)
Cladribine	7 (0·0%)	0	5 (0·0%)	1 (0·0%)
IL-1 inhibitor (canakinumab, anakinra, or rilonacept)	9 (0·0%)	0	8 (0·0%)	1 (0·0%)
IL-6 inhibitor (sarilumab, tocilizumab, or satralizumab)	96 (0·0%)	11 (0·0%)	79 (0·0%)	15 (0·0%)
IL-12/23 inhibitor (ustekinumab)	271 (0·0%)	22 (0·0%)	178 (0·0%)	27 (0·0%)
IL-17 inhibitor (ixekizumab, brodalumab, or secukinumab)	271 (0·0%)	32 (0·0%)	187 (0·0%)	34 (0·0%)
IL-23 inhibitor (guselkumab, tildrakizumab, or risankizumab)	54 (0·0%)	10 (0·0%)	69 (0·0%)	8 (0·0%)
Abatacept	115 (0·0%)	14 (0·0%)	83 (0·0%)	28 (0·0%)
Anti-BlyS (belimumab)	34 (0·0%)	0	35 (0·0%)	7 (0·0%)
S1P receptor modulator (siponimod, ponesimod, fingolimod, or ozanimod)	73 (0·0%)	7 (0·0%)	49 (0·0%)	13 (0·0%)
JAK inhibitor (tofacitinib, upadacitinib, or baricitinib)	304 (0·0%)	46 (0·0%)	266 (0·0%)	48 (0·0%)
Integrin inhibitor (edolizumab or natalizumab)	38 (0·0%)	0	23 (0·0%)	5 (0·0%)
Targeted synthetic PDE4 inhibitor (apremilast)	167 (0·0%)	15 (0·0%)	129 (0·0%)	13 (0·0%)
Anti-CD20 (rituximab, ocrelizumab, or ofatumumab)	67 (0·0%)	12 (0·0%)	51 (0·0%)	16 (0·0%)
Budesonide	2142 (0·1%)	217 (0·1%)	1429 (0·2%)	256 (0·2%)
Systemic glucocorticoid (prednisone, dexamethasone, prednisolone, triamcinolone, methylprednisolone, or hydrocortisone)	56 506 (3·7%)	7596 (4·5%)	39 670 (6·1%)	8687 (7·2%)

Data are n (%) or median (IQR). IMID=immune-mediated inflammatory disease. IMM=immunomodulatory medication. NA=not applicable.

* Total patient number exluding patients without previous history at Providence St Joseph Health.

† Some patients had more than one IMID diagnosis.

**Table 2:** Severe COVID-19 outcomes in patients with and without IMIDs during the pre-omicron and omicron-predominant periods

	Tested for COVID-19	Positive for COVID-19*	p value	Hospitalised†	p value	Received mechanical ventilation†	p value	Died†	p value
**Pre-omicron period (March 1, 2020–Dec 25, 2021)**									
Total patients‡	1 517 295 (100·0%)	169 993 (11·2%)	··	23 330 (13·7%)	··	1072 (0·6%)	··	5294 (3·1%)	··
Without IMIDs§	1 433 798 (94·5%)	161 923 (11·3%)	··	22 154 (13·7%)	··	1021 (0·6%)	··	4980 (3·1%)	··
With IMIDs§	83 497 (5·5%)	8070 (9·7%)	<0·0001	1176 (14·6%)	<0·024	51 (0·6%)	0·94	314 (3·9%)	<0·0001
**Omicron-predominant period (Dec 26, 2021–Aug 30, 2022)**									
Total patients‡	650 361 (100·0%)	120 862 (18·6%)	··	14 504 (12·0%)	··	567 (0·5%)	··	2001 (1·7%)	··
Without IMIDs§	608 112 (93·5%)	113 535 (18·7%)	··	13 422 (11·8%)	··	531 (0·5%)	··	1814 (1·6%)	··
With IMIDs§	42 249 (6·5%)	7327 (17·3%)	<0·0001	1082 (14·8%)	<0·0001	36 (0·5%)	0·72	187 (2·6%)	<0·0001

Data are n (%), unless otherwise indicated. IMID=immune-mediated inflammatory disease. PSJH=Providence St Joseph Health.

* Proportion of individuals tested for COVID-19.

† Proportion of patients with COVID-19.

‡ Total patient number exluding patients without previous history at PSJH.

§ IMIDs (excluding asthma): rheumatoid arthritis, spondyloarthritis, systemic lupus erythematosus, psoriatic arthritis, systemic sclerosis, vasculitis, sarcoidosis, antiphospholipid syndrome, Sjögren’s syndrome, inflammatory bowel disease, multiple sclerosis, and psoriasis.

The p values for the comparison between the groups with and without IMIDs (categorical variables) are calculated with Fisher’s exact test.

## Data Availability

All code associated with the models has been shared. Results have been aggregated and reported within this Article to the maximum extent possible, while maintaining privacy from personal health information as required by law. All data are archived within PSJH systems in an audited computing environment secured by the Health Insurance Portability and Accountability Act to facilitate verification of study conclusions. The code for extracting, cleaning, and analysing the data in this Article is available on GitHub (https://github.com/Hadlock-Lab/Risk_factors_for_severe_COVID19_outcomes_a_study_of_IMIDs_medications_comorbidities).

## Research in context

### Evidence before this study

We aimed to understand severe COVID-19 outcomes and to investigate the effects of the pandemic time period and the risks associated with individual immune-mediated inflammatory diseases (IMIDs), classes of immunomodulatory medications (IMMs), chronic comorbidities, and COVID-19 vaccination status. We searched PubMed, medRxiv, bioRxiv, and Google Scholar for large-population, multivariable studies published in English from Jan 1, 2020, to April 24, 2023, using the following search terms: “COVID-19” with “immune-mediated inflammatory disease”, “autoimmune disease”, “immunomodulatory medication”, “immunosuppressive medication”, and “rheumatic disease”, as well as “infection”, “hospitalisation”, “mechanical ventilation”, and “mortality” risk. We also searched for rates related to COVID-19 in patients receiving IMMs. To our knowledge, as of April 24, 2023, no study has investigated the risk of severe COVID-19 outcomes accounting for the interconnected factors of specific IMIDs, classes of IMMs, chronic comorbidities, and COVID-19 vaccination status, with consideration of SARS-CoV-2 variants dominant in the early pandemic period and more recent omicron variants.

### Added value of this study

We developed high-performance machine learning models on data from electronic health records for over 2 million patients who were tested for COVID-19 across seven states in the USA at two different timeframes across the pandemic: the pre-omicron period (March 1, 2020–Dec 25, 2021) and the omicron-predominant period (Dec 26, 2021–Aug 30, 2022). In both timeframes, patients with IMIDs had decreased rates of COVID-19; however, individuals with both an IMID and COVID-19 had an increased rate of hospitalisation and mortality. Age and chronic comorbidities were associated with worse outcomes, whereas vaccinations and boosters were associated with improved outcomes in all patients with COVID-19. Selected IMIDs showed an increased risk of severe COVID-19 outcomes; however, asthma, psoriasis, and spondyloarthritis were associated with better outcomes than were expected for the overall population. Most classes of IMMs were not associated with an increased risk of severe COVID-19 outcomes, but some IMMs were limited by sample size and require further study.

### Implications of all the available evidence

Although patients with IMIDs are at an increased risk of severe COVID-19 outcomes, this research suggests that age, comorbidities, and not being fully vaccinated are greater risk factors than the IMID diagnoses themselves or the use of IMMs. The finding that asthma, psoriasis, and spondyloarthritis were associated with better outcomes than those expected for the overall population suggests that some IMIDs might provide new insight into protective mechanisms against COVID-19. Overall, there is a need to take age and comorbidities into consideration when developing COVID-19 guidelines for patients with IMIDs. In addition, further research is needed for each specific IMID (with inclusion of IMID severity at the time of COVID-19 infection) and each IMM (with consideration of pre-COVID-19 dosage and timing).
