# Supplementary material for: Machine learning to understand risks for severe COVID-19 outcomes: a retrospective cohort study of immune-mediated inflammatory diseases, immunomodulatory medications, and comorbidities in a large US health-care system
Source: Lancet Digit Health. Author manuscript; Available in PMC 2024 May 4. (PMC11069366; doi:10.1016/S2589-7500(24)00021-9)
Supplement: 1 [file NIHMS1989112-supplement-1.pdf]

THE LANCET
Digital Health

Supplementary appendix

This appendix formed part of the original submission and has been peer reviewed. We post it as supplied by the authors.

Supplement to: Wei Q, Mease PJ, Chiorean M, et al. Machine learning to understand risks for severe COVID-19 outcomes: a retrospective cohort study of immune-mediated inflammatory diseases, immunomodulatory medications, and comorbidities in a large US health-care system. *Lancet Digit Health* 2024; **6:** e309–22.

Supplementary Material for

Machine learning to understand risks for severe COVID-19 outcomes: a retrospective cohort study of immunemediated inflammatory diseases, immunomodulatory medications, and comorbidities in a large US health-care system

Table of Contents

Supplementary Figures 2

Supplementary Figure S1. Machine learning models performance on the 10% hold out test set. 2

Supplementary Figure S2. Feature importance for all extreme gradient boosting models 3

Supplementary Figure S3. Feature importance for all features of all-age model of hospitalisation within the window of three days prior to 14 days of their first COVID-19 incidence, or MV or death within 30 days of their first COVID-19 incidence across Time Interval 1 (01/03/2020 to 25/12/2021) and Time Interval 2 (26/12/2021 to 30/08/2022)...4

Supplementary Figure S4. Feature importance for all features of all-age model of MV or death within 30 days of their first COVID-19 incidence across Time Interval 1 (01/03/2020 to 25/12/2021) and Time Interval 2 (26/12/2021 to 30/08/2022). 5

Supplementary Figure S5. Feature importance for all features of all-age model of death within 30 days of their first COVID-19 incidence across Time Interval 1 (01/03/2020 to 25/12/2021) and Time Interval 2 (26/12/2021 to 30/08/2022)...6

Supplementary Figure S6. Log transformed adjusted odds ratio of logistic regression models, including anti-SARS-CoV-2 neutralizing monoclonal antibody factor. 7

Supplementary Figure S7. Log transformed adjusted odds ratio of logistic regression models, excluding patients treated with anti-SARS-CoV-2 neutralizing monoclonal antibodies...8

Supplementary Tables 10

Supplementary Table S1. XGB classification algorithm hyperparameters and hyperparameter range used in random-search tuning. 10

Supplementary Table S2. Results of the multivariable logistic regression (LR) model, Selected factors for hospitalisation, MV, or death at Time Interval 1 (01/03/2020 to 25/12/2021) in COVID-19 positive patients. 10

Supplementary Table S3. Results of the multivariable logistic regression (LR) model, Selected factors for hospitalisation, MV, or death at Time Interval 2 (26/12/2021 to 30/08/2022) in COVID-19 positive patients. 11

Supplementary Table S4. Results of the multivariable logistic regression (LR) model, Selected factors for MV or death at Time Interval 1 (01/03/2020 to 25/12/2021) in COVID-19 positive patients. 13

Supplementary Table S5. Results of the multivariable logistic regression (LR) model, Selected factors for MV or death at Time Interval 2 (26/12/2021 to 30/08/2022) in COVID-19 positive patients. 14

Supplementary Table S6. Results of the multivariable logistic regression (LR) model, Selected factors for death at Time Interval 1 (01/03/2020 to 25/12/2021) in COVID-19 positive patients..15

Supplementary Table S7. Results of the multivariable logistic regression (LR) model, Selected factors for death at Time Interval 2 (26/12/2021 to 30/08/2022) in COVID-19 positive patients..17

Supplementary Table S8. SNOMED definitions of IMIDs in this paper...18

Supplementary Table S9. Specific medications of each immunomodulatory drug class and monoclonal antibody class in this paper...20

Supplementary Table S10. TRIPOD Statement...21

Supplementary Table S11. STROBE Statement...23

Supplementary Table S12. Classification Machine learning algorithms hyperparameters and hyperparameter values used in performance evaluation. 27

Supplementary Discussion 28

Supplementary Discussion S1. Anti-SARS-CoV-2 neutralizing monoclonal antibodies (NmAbs) analysis. 28

Supplementary Discussion S2. Nirmatrelvir-lopinavir/ritonavir analysis. 28

Supplementary References.. 28

Supplementary Figures

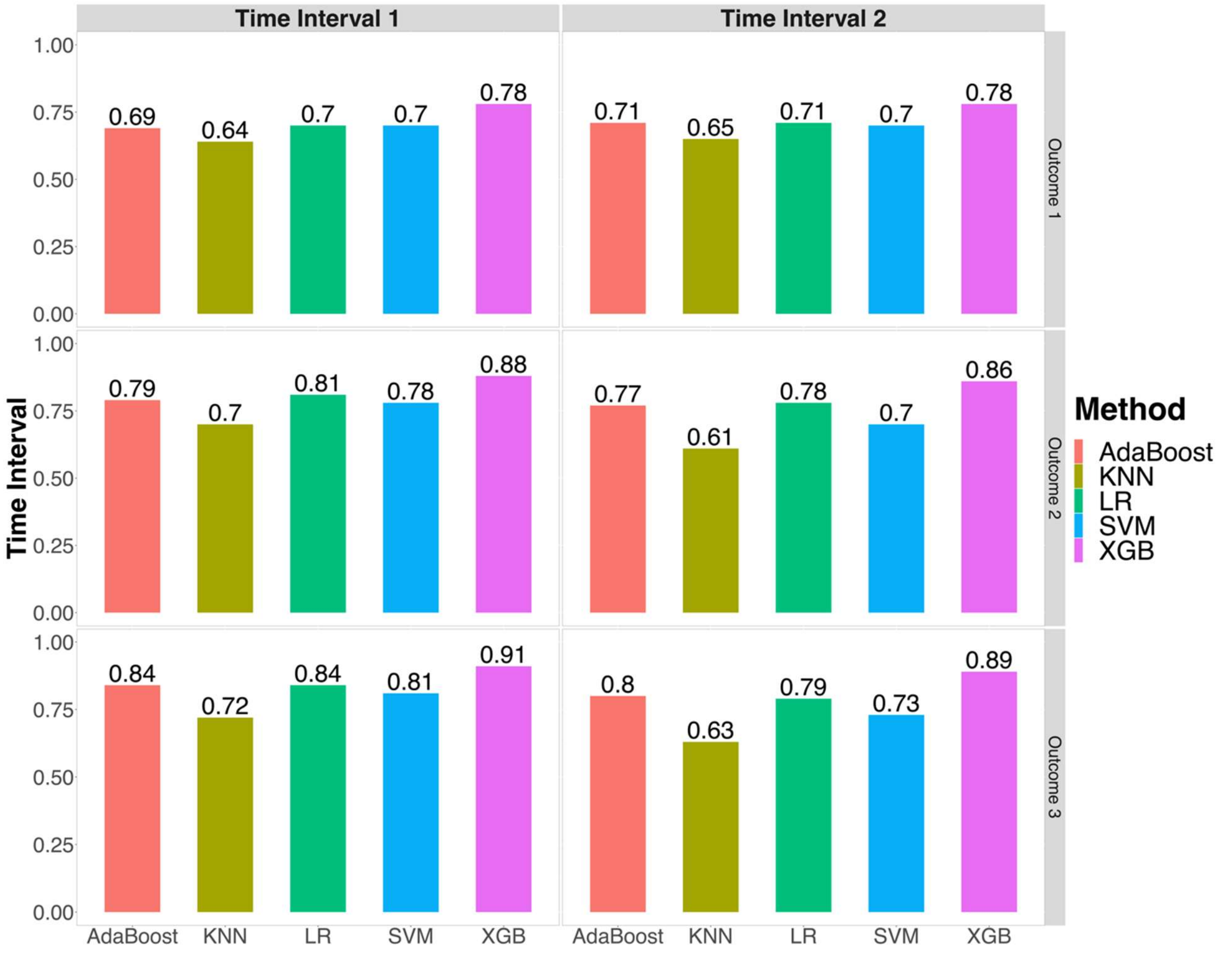

Supplementary Figure S1. Machine learning models performance on the 10% hold out test set. Performance for machine learning models for severe outcomes (1. hospitalisation or MV or death, 2. MV or death, and 3. death) across Time Interval 1 (01/03/2020 to 25/12/2021) and Time Interval 2 (26/12/2021 to 30/08/2022)· AUROC = area under the receiver-operator characteristic curve, LR = logistic regression, XGB = gradient boosting, AdaBoost = Adaptive Boosting, KNN = K-nearest neighbours, and SVM = Support Vector Machine. Details of each models' hyperparameters' name and values can be found in Supplementary Table S1 and S12.

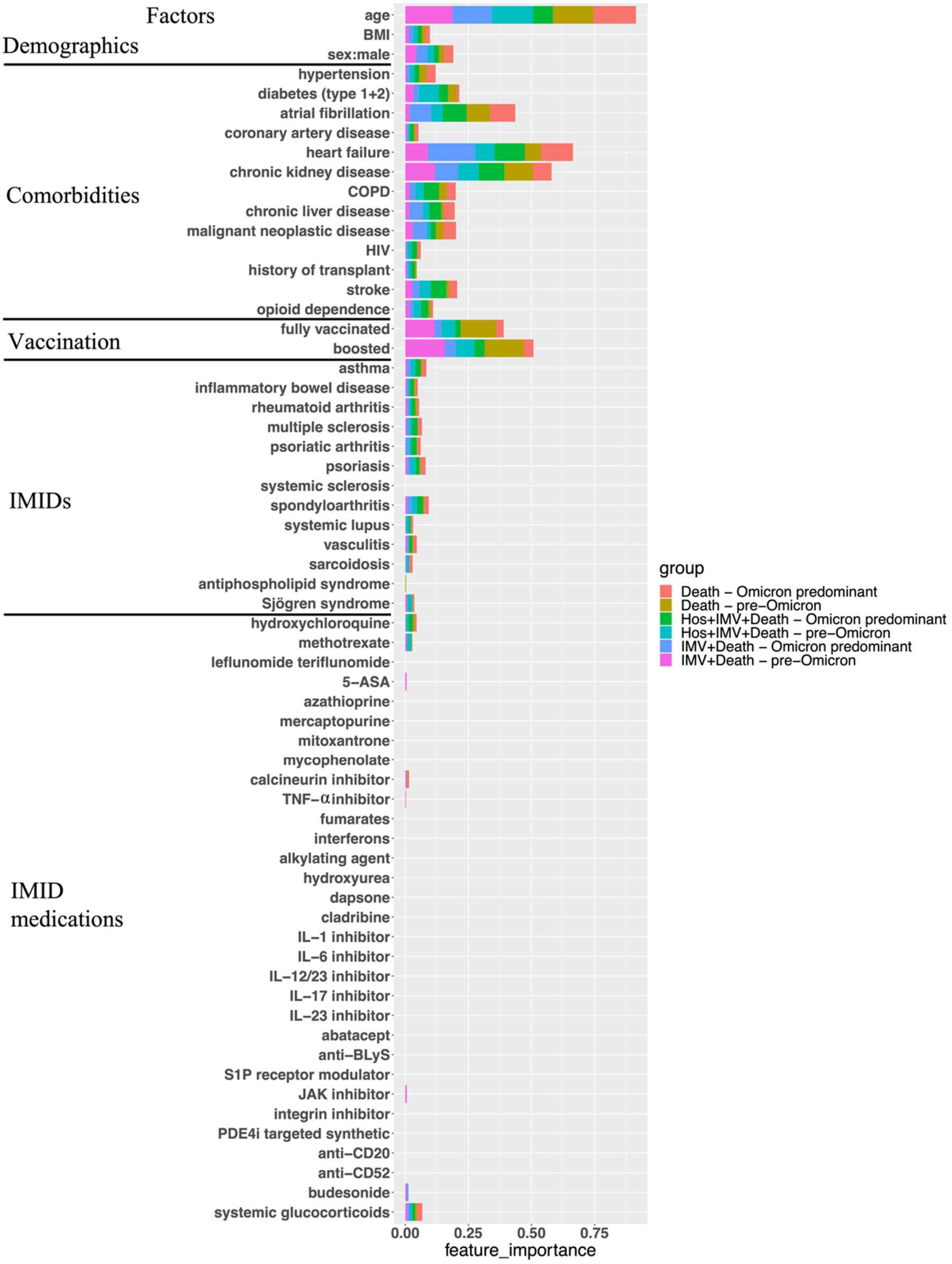

Supplementary Figure S2. Feature importance for all extreme gradient boosting models

Feature importance for all 63 factors for XGB models for hospitalisation and death across Time Interval 1 (01/03/2020 to 25/12/2021) and Time Interval 2 (26/12/2021 to 30/08/2022) using six different colours (coral, brown, green, cyan, blue, and purple). The feature importance of each feature is calculated using the number of times a feature is used to split the data across all trees.

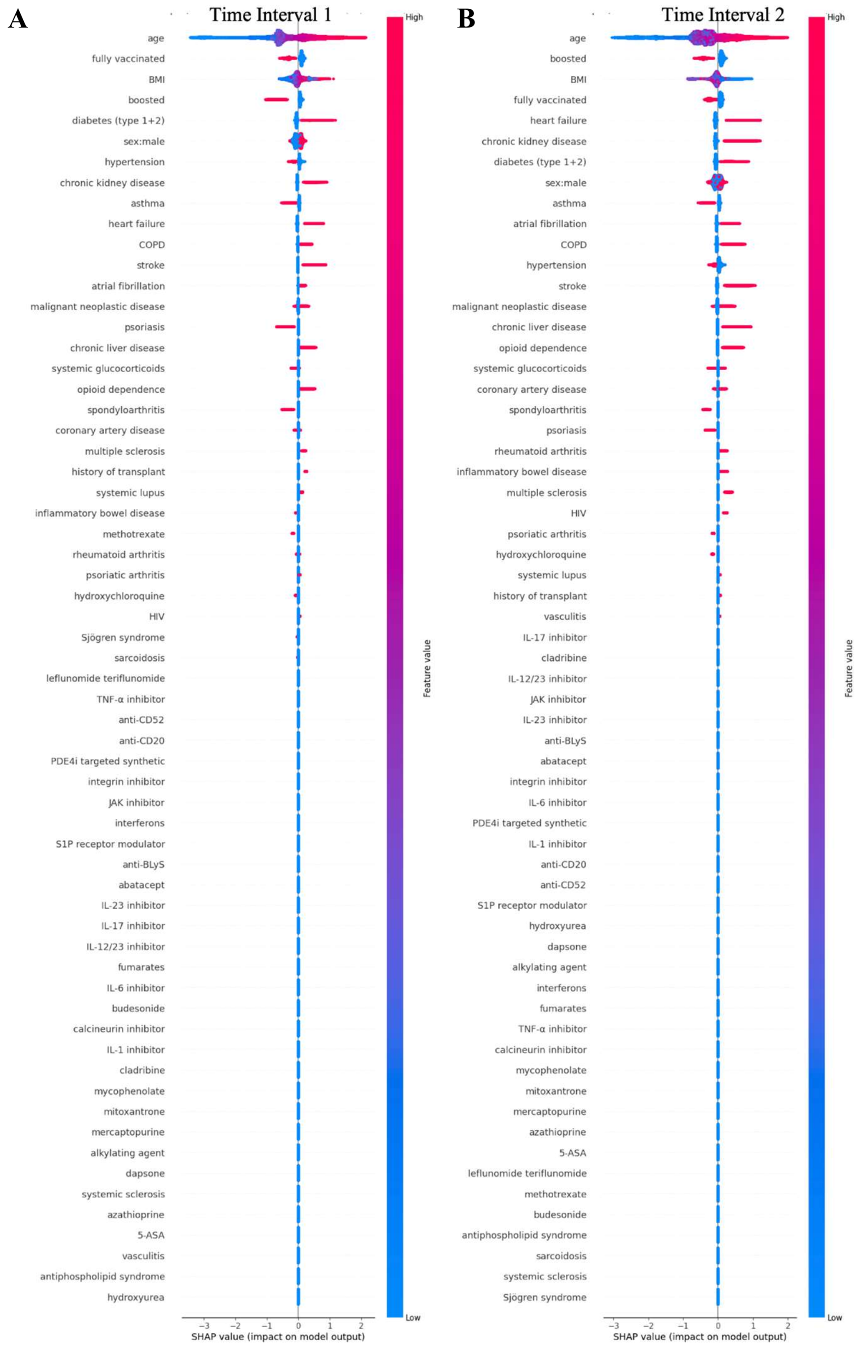

Supplementary Figure S3. Feature importance for all features of all-age model of hospitalisation within the window of three days prior to 14 days of their first COVID-19 incidence, or MV or death within 30 days of their first COVID-19 incidence across Time Interval 1 (01/03/2020 to 25/12/2021) and Time Interval 2 (26/12/2021 to 30/08/2022).

Extreme gradient boosting feature importance and influence of higher and lower values of the factors on the all-age group population outcome. The left side of each plot is associated with reduced risk and the right side of each plot is associated with increased risk. Red dots represent patients with higher values for a variable, and blue dots are patients with lower values. Nominal classes are binary [0, 1]. For sex, male is 1 (red).

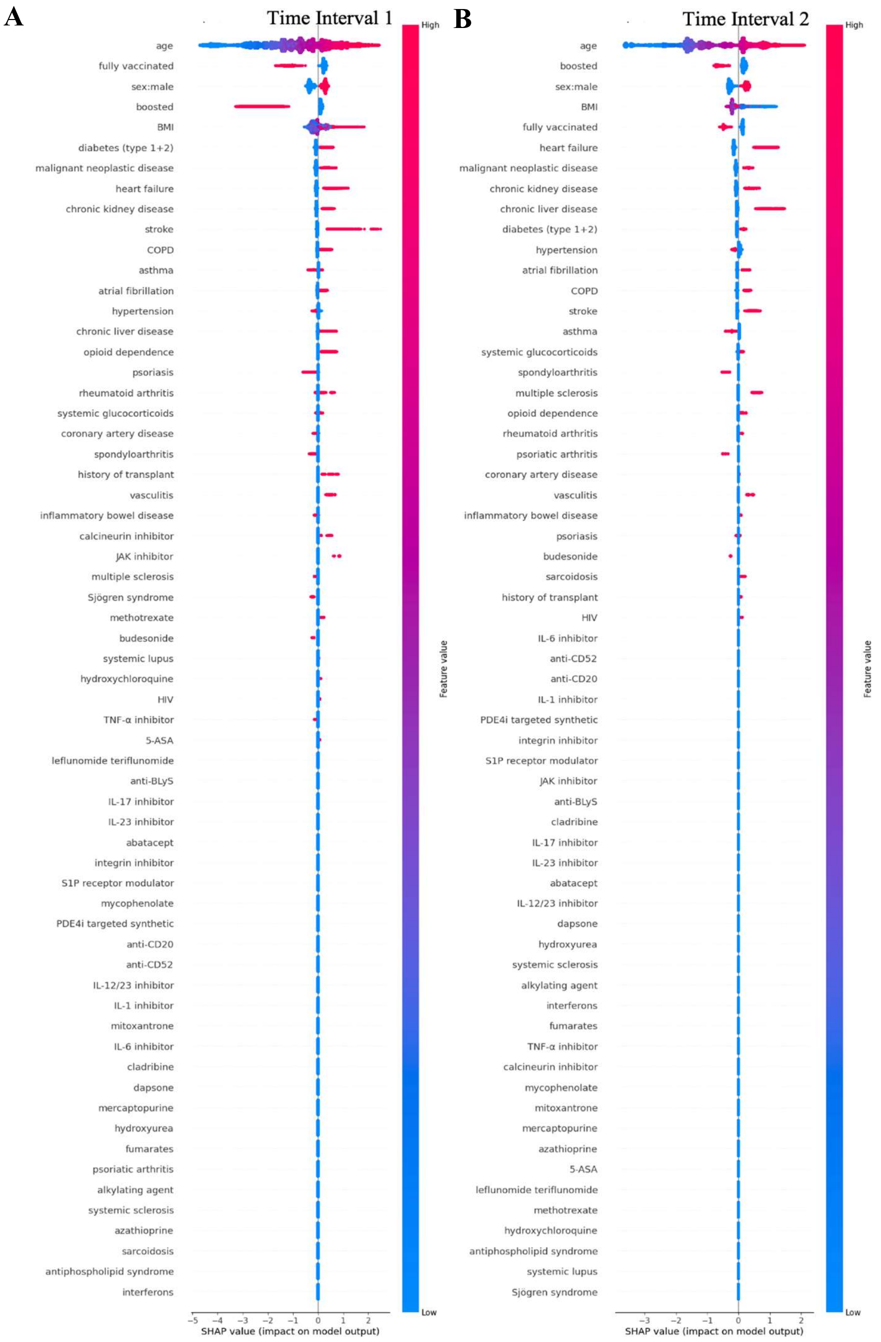

Supplementary Figure S4. Feature importance for all features of all-age model of MV or death within 30 days of their first COVID-19 incidence across Time Interval 1 (01/03/2020 to 25/12/2021) and Time Interval 2 (26/12/2021 to 30/08/2022).

Extreme gradient boosting feature importance and influence of higher and lower values of the factors on the all-age group population outcome. In both A and B plots, SHAP values < 0 are associated with reduced risk for mechanical ventilation or death, and SHAP values > 0 are associated with increased risk. Red dots represent patients with higher values for a variable, and blue dots are patients with lower values· Nominal classes are binary [0, 1]. For sex, male is 1 (red).

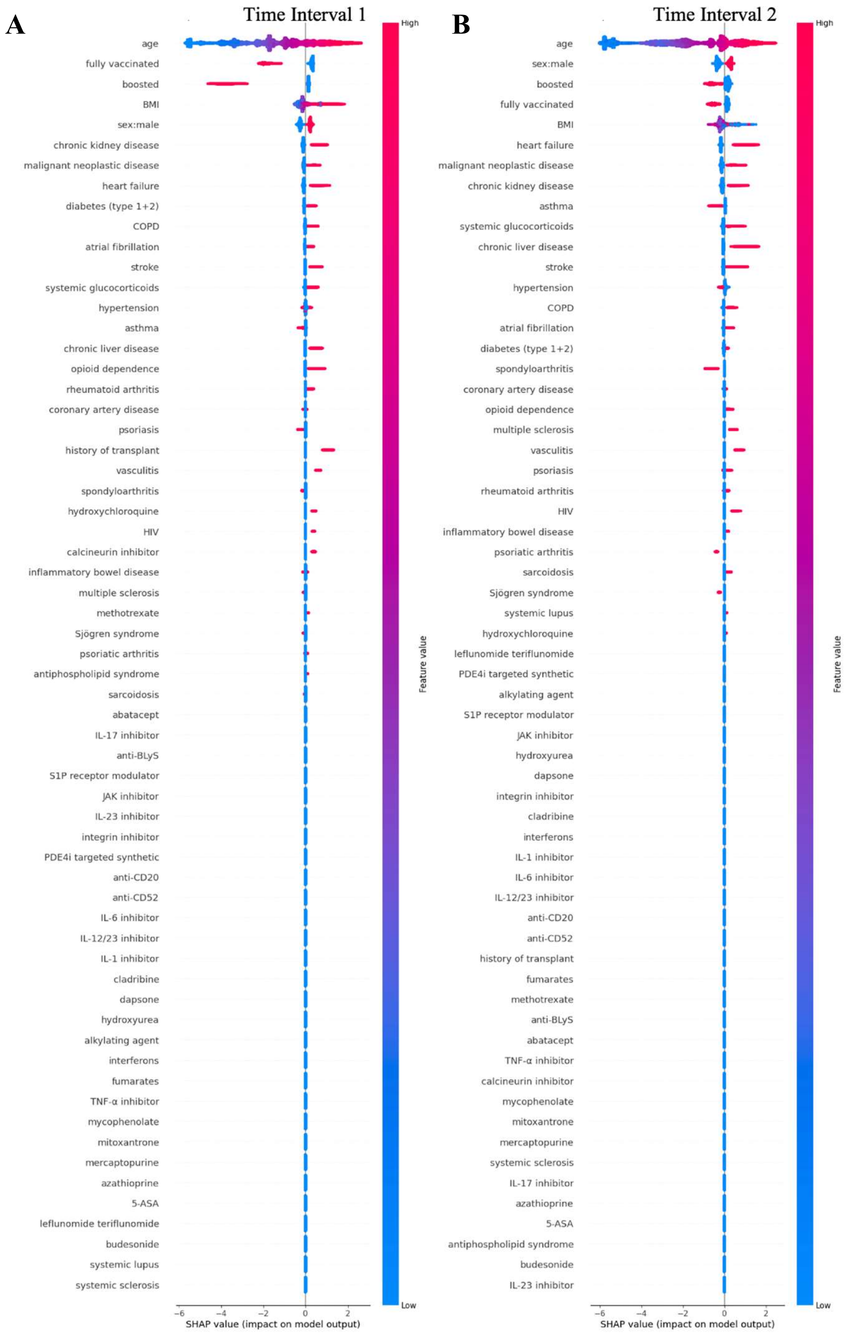

Supplementary Figure S5. Feature importance for all features of all-age model of death within 30 days of their first COVID-19 incidence across Time Interval 1 (01/03/2020 to 25/12/2021) and Time Interval 2 (26/12/2021 to 30/08/2022).

Extreme gradient boosting feature importance and influence of higher and lower values of the factors on the all-age group population outcome. In both A and B plots, SHAP values < 0 are associated with reduced risk for mechanical ventilation or death, and SHAP values > 0 are associated with increased risk. Red dots represent patients with higher values for a variable, and blue dots are patients with lower values. Nominal classes are binary [0, 1]. For sex, male is 1 (red).

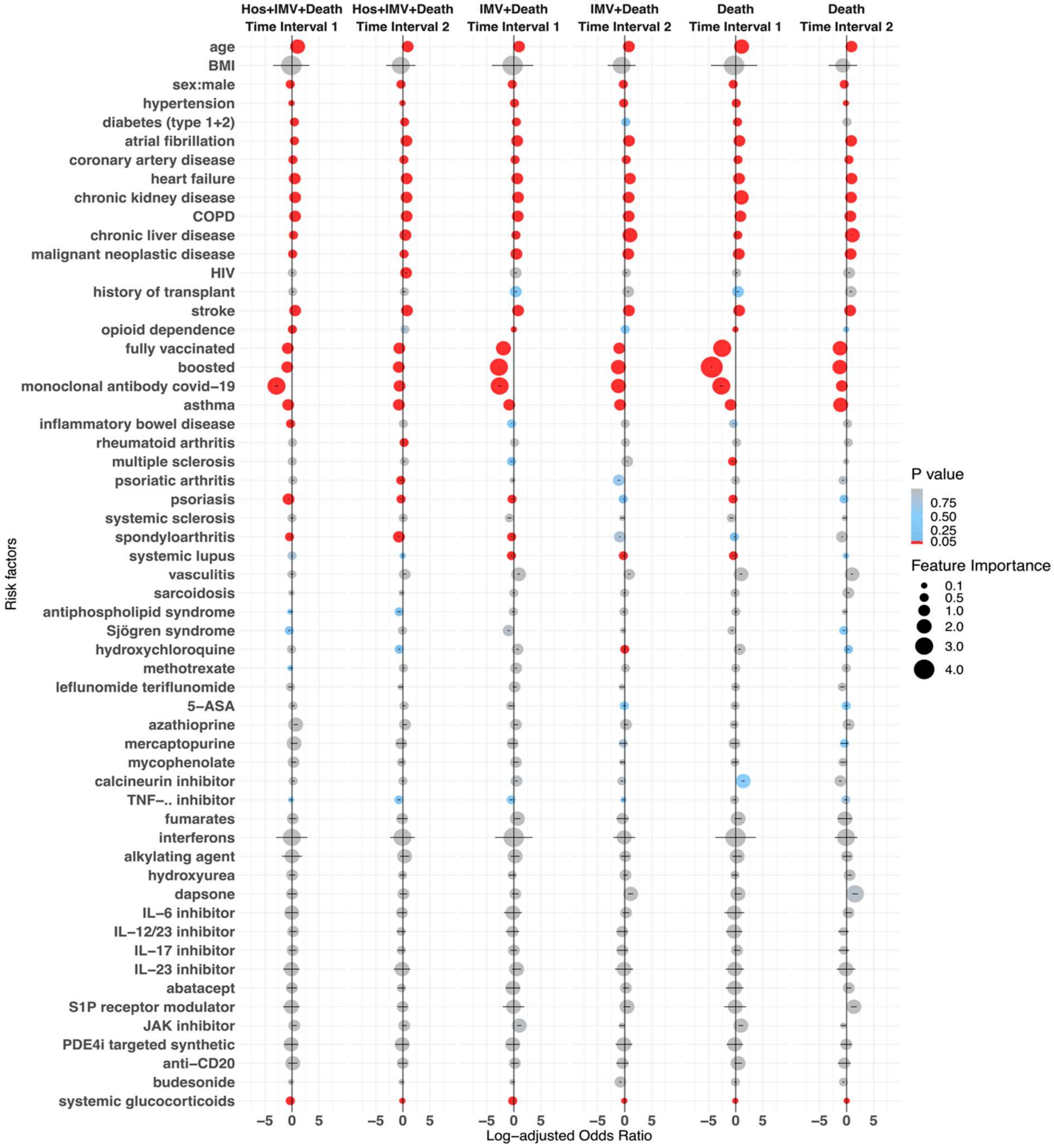

Supplementary Figure S6. Log transformed adjusted odds ratio of logistic regression models, including anti-SARS-CoV-2 neutralizing monoclonal antibody factor.

Selected factors for hospitalisation, MV or death, MV or death, and death across Time Interval 1 (01/03/2020 to 25/12/2021) and Time Interval 2 (26/12/2021 to 30/08/2022) in COVID-19 positive patients, using multivariable logistic regression (LR). The colour of dots represent P-value calculated using raw data. Position and size of dots represents log-adjusted odds ratio and feature importance from over-sampling. The error bar represents the 95% confidence interval. Factors with fewer than ten observations were excluded.

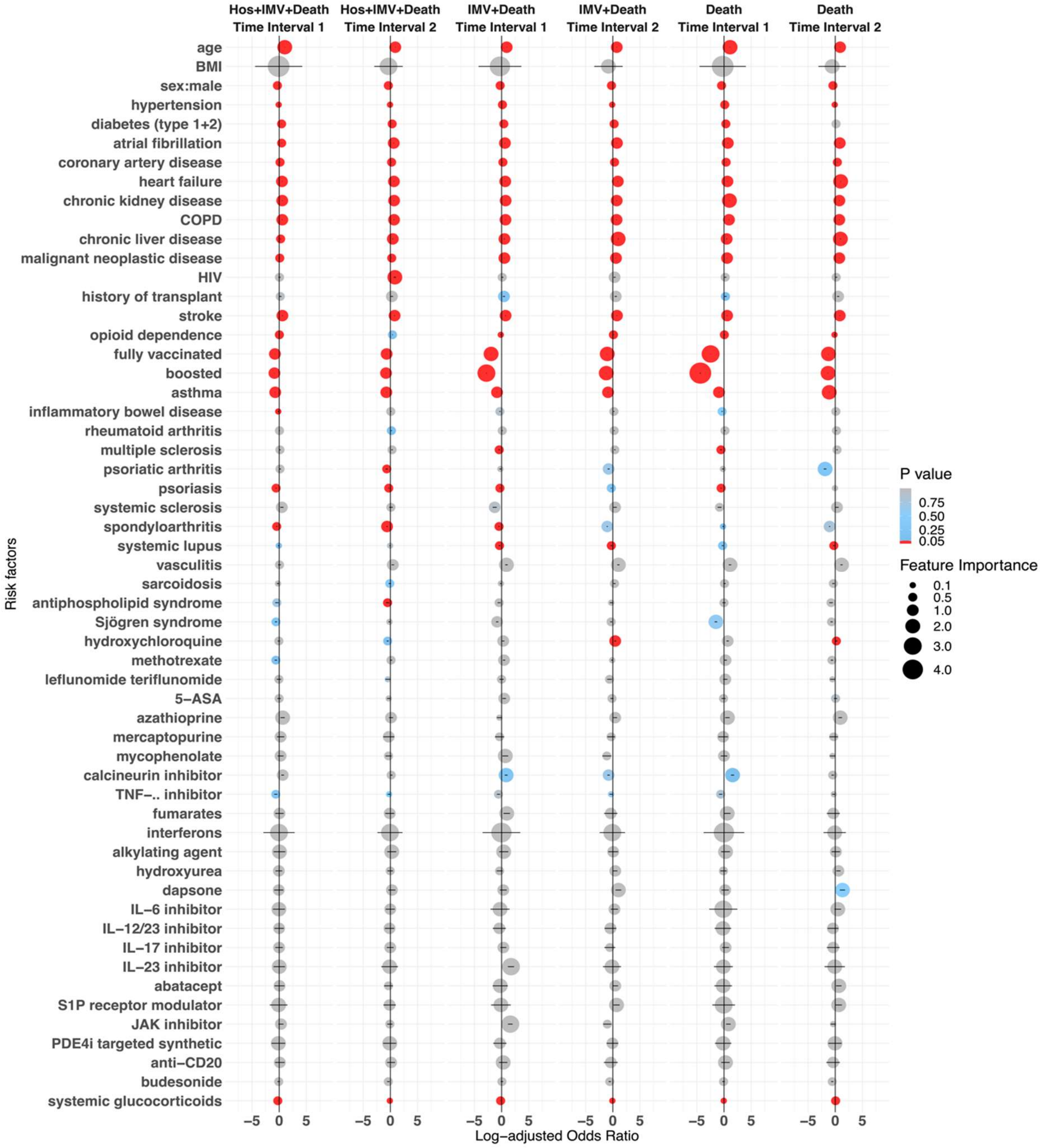

Supplementary Figure S7. Log transformed adjusted odds ratio of logistic regression models, excluding patients treated with anti-SARS-CoV-2 neutralizing monoclonal antibodies.
Selected factors for hospitalisation, MV or death, MV or death, and death across Time Interval 1 (01/03/2020 to 25/12/2021) and Time Interval 2 (26/12/2021 to 30/08/2022) in COVID-19 positive patients, using multivariable logistic regression (LR). The colour of dots represent P-value calculated using raw data. Position and size of dots represents log-adjusted odds ratio and feature importance from over-sampling. The error bar represents the 95% confidence interval. Factors with fewer than ten observations were excluded.

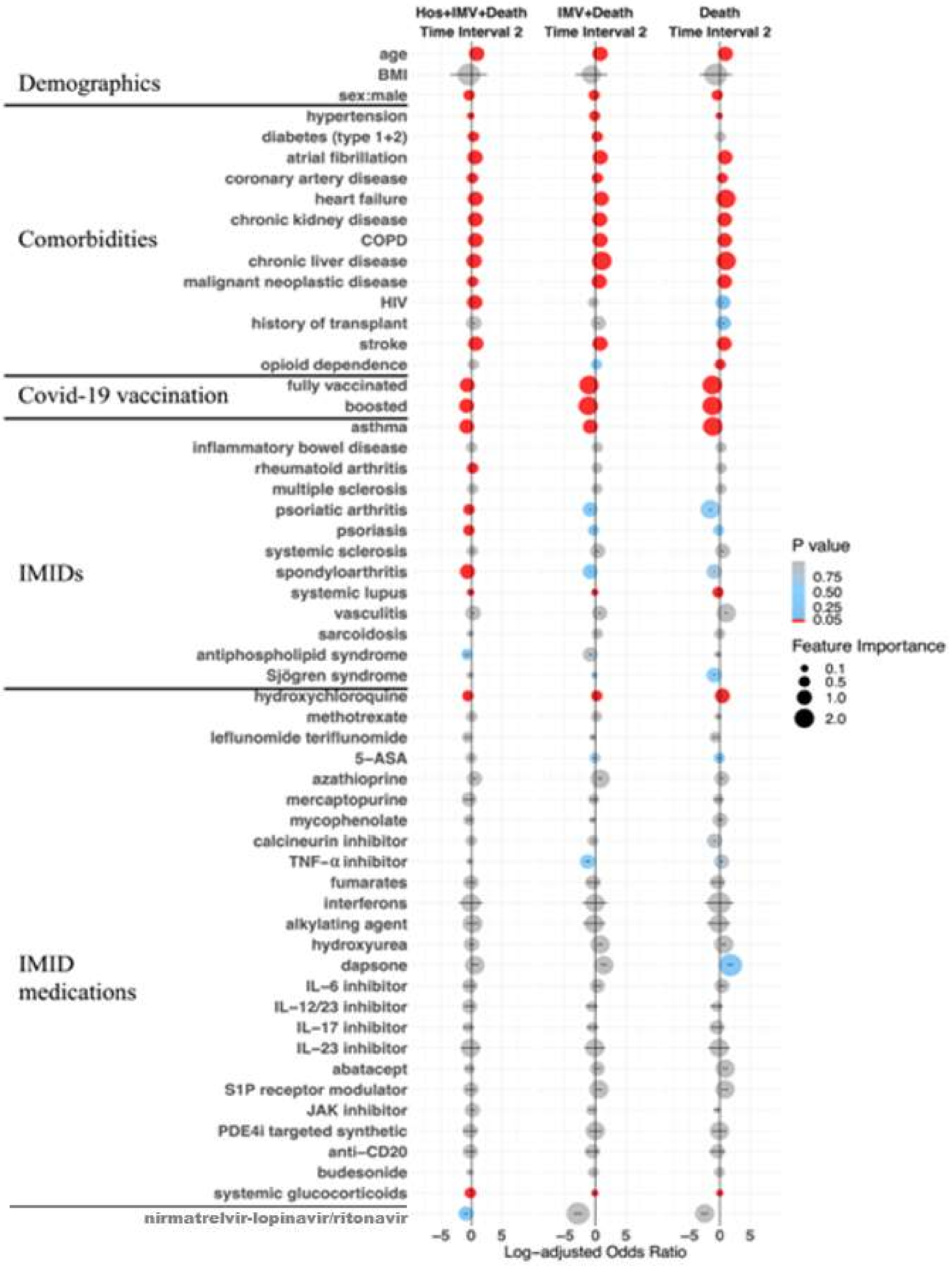

Supplementary Figure S8. Log transformed adjusted odds ratio of logistic regression models, including the usage of nirmatrelvir-lopinavir/ritonavir as an additional risk factor. Selected factors for hospitalisation, MV or death, MV or death, and death Time Interval 2 (26/12/2021 to 30/08/2022) in COVID-19 positive patients, using multivariable logistic regression (LR). The colour of dots represent P-value calculated using raw data. Position and size of dots represents log-adjusted odds ratio and feature importance from over-sampling. The error bar represents the 95% confidence interval. Factors with fewer than ten observations were excluded.

Supplementary Tables

Supplementary Table S1. XGB classification algorithm hyperparameters and hyperparameter range used in random-search tuning.

Iteration number set to 1500.

Name	Description	Range
learning_rate	boosting learning rate	(0·01, 0·05, 0·1, 0·2)
n_estimators	number of trees to fit	(5, 10, 15, …, 200)
max_depth	maximum tree depth	(2, 3, 5, …, 15)
min_child_weight	minimum sum of instance weight needed in a child	(0, 1, 2, ···, 50)
subsample	sub-sample ratio of the training instance	(0·4, 0·5, 0·6, …, 1·0)
colsample_bytree	sub-sample ratio of columns when constructing each tree	(0·4, 0·5, 0·6, …, 1·0)
reg_alpha	coefficient of L1 regularization for the node weights	(0, 1, 2, 3, 4, 5)
reg_lambda	coefficient of L2 regularization for the node weights	(1, 2, 3, 4, 5)
scale_pos_weight	control the balance of positive and negative weights, useful for unbalanced classes·	sum(negative instances) / sum(positive instances)
importance_type	define tree model importance type	('weight')
random_state	random number seed	(42)

Supplementary Table S2. Results of the multivariable logistic regression (LR) model, Selected factors for hospitalisation, MV, or death at Time Interval 1 (01/03/2020 to 25/12/2021) in COVID-19 positive patients.

Feature	Adjusted odds ratio (AOR)	95% confidence interval (low)	95% confidence interval (high)	Log of AOR	Feature importance	P value	P value (FDR corrected)
age	2.776	2.702	2.852	1.021	1.048	< 0.0001	< 0.0001
BMI	0.888	0.032	24.754	-0.118	3.209	0.920	1.000
sex: male	0.735	0.723	0.746	-0.308	0.293	< 0.0001	< 0.0001
hypertension	0.902	0.882	0.924	-0.103	0.079	< 0.0001	< 0.0001
diabetes (type 1+2)	1.543	1.502	1.586	0.434	0.461	< 0.0001	< 0.0001
atrial fibrillation	1.553	1.492	1.616	0.440	0.480	< 0.0001	< 0.0001
coronary artery disease	1.178	1.133	1.224	0.164	0.202	< 0.0001	< 0.0001
heart failure	1.678	1.610	1.749	0.518	0.559	< 0.0001	< 0.0001
chronic kidney disease	1.781	1.718	1.846	0.577	0.613	< 0.0001	< 0.0001
COPD	1.779	1.704	1.857	0.576	0.619	< 0.0001	< 0.0001
chronic liver disease	1.337	1.250	1.432	0.291	0.359	< 0.0001	< 0.0001
malignant neoplastic disease	1.127	1.095	1.160	0.120	0.148	< 0.0001	< 0.0001
HIV	1.107	0.914	1.340	0.101	0.293	0.920	1.000
history of transplant	1.180	0.944	1.476	0.165	0.389	0.201	1.000
stroke	1.780	1.682	1.885	0.577	0.634	< 0.0001	< 0.0001
opioid dependence	1.079	1.007	1.155	0.076	0.144	0.001	0.013
fully vaccinated	0.459	0.450	0.468	-0.779	0.760	< 0.0001	< 0.0001
boosted	0.420	0.407	0.433	-0.869	0.838	< 0.0001	< 0.0001
asthma	0.493	0.479	0.509	-0.707	0.676	< 0.0001	< 0.0001
inflammatory bowel disease	0.776	0.702	0.857	-0.254	0.154	0.001	0.013

rheumatoid arthritis	1.098	1.016	1.186	0.094	0.171	0.499	1.000
multiple sclerosis	1.044	0.914	1.192	0.043	0.176	0.589	1.000
psoriatic arthritis	1.367	1.156	1.616	0.312	0.480	0.455	1.000
psoriasis	0.519	0.478	0.564	-0.656	0.572	< 0.0001	< 0.0001
systemic sclerosis	0.994	0.729	1.355	-0.006	0.304	0.826	1.000
spondyloarthritis	0.642	0.583	0.707	-0.444	0.347	< 0.0001	< 0.0001
systemic lupus	0.990	0.867	1.132	-0.010	0.124	0.042	0.452
vasculitis	0.942	0.733	1.210	-0.060	0.191	0.215	1.000
sarcoidosis	0.875	0.719	1.064	-0.134	0.062	0.297	1.000
antiphospholipid syndrome	0.705	0.528	0.942	-0.349	0.060	0.038	0.427
Sjögren's syndrome	0.520	0.425	0.635	-0.655	0.454	0.007	0.086
hydroxychloroquine	0.869	0.704	1.071	-0.141	0.069	0.162	1.000
methotrexate	0.677	0.543	0.845	-0.389	0.168	0.009	0.106
leflunomide teriflunomide	0.771	0.503	1.181	-0.260	0.166	0.736	1.000
5-ASA	1.227	0.906	1.660	0.204	0.507	0.806	1.000
azathioprine	1.980	1.340	2.924	0.683	1.073	0.155	1.000
mercaptopurine	1.464	0.773	2.773	0.381	1.020	0.896	1.000
mycophenolate	1.372	0.818	2.300	0.316	0.833	0.923	1.000
calcineurin inhibitor	1.340	1.037	1.733	0.293	0.550	0.879	1.000
TNF-α inhibitor	0.818	0.618	1.082	-0.201	0.079	0.048	0.496
fumarates	1.137	0.568	2.277	0.128	0.823	0.928	1.000
interferons	0.970	0.054	17.532	-0.030	2.864	0.994	1.000
alkylating agent	1.001	0.160	6.253	0.001	1.833	0.982	1.000
hydroxyurea	1.037	0.584	1.839	0.036	0.609	0.785	1.000
dapsone	1.018	0.433	2.392	0.018	0.872	0.970	1.000
IL-6 inhibitor	0.956	0.300	3.047	-0.045	1.114	0.947	1.000
IL-12/23 inhibitor	1.208	0.598	2.440	0.189	0.892	0.940	1.000
IL-17 inhibitor	1.099	0.567	2.128	0.094	0.755	0.989	1.000
IL-23 inhibitor	0.893	0.193	4.129	-0.113	1.418	0.940	1.000
abatacept	0.978	0.399	2.399	-0.022	0.875	0.965	1.000
S1P receptor modulator	0.889	0.194	4.067	-0.118	1.403	0.896	1.000
JAK inhibitor	1.519	0.969	2.380	0.418	0.867	0.376	1.000
PDE4i targeted synthetic	0.857	0.244	3.007	-0.154	1.101	0.889	1.000
anti-CD20	1.144	0.481	2.718	0.134	1.000	0.873	1.000
budesonide	0.808	0.640	1.020	-0.213	0.020	0.344	1.000
systemic glucocorticoids	0.714	0.686	0.744	-0.337	0.296	< 0.001	< 0.001

Supplementary Table S3. Results of the multivariable logistic regression (LR) model, Selected factors for hospitalisation, MV, or death at Time Interval 2 (26/12/2021 to 30/08/2022) in COVID-19 positive patients.

Feature	Adjusted odds ratio (AOR)	95% confidence interval (low)	95% confidence interval (high)	Log of AOR	Feature importance	P value	P value (FDR corrected)
age	2.357	2.277	2.440	0.857	0.892	< 0.0001	< 0.0001
BMI	0.698	0.047	10.340	-0.359	2.336	0.597	1.000
sex: male	0.700	0.687	0.713	-0.357	0.338	< 0.0001	< 0.0001
hypertension	0.919	0.895	0.944	-0.084	0.058	< 0.0001	< 0.0001
diabetes (type 1+2)	1.373	1.332	1.416	0.317	0.348	< 0.0001	< 0.0001

atrial fibrillation	1.863	1.791	1.939	0.622	0.662	< 0.0001	< 0.0001
coronary artery disease	1.189	1.143	1.237	0.173	0.213	< 0.0001	< 0.0001
heart failure	1.977	1.898	2.059	0.682	0.722	< 0.0001	< 0.0001
chronic kidney disease	1.935	1.866	2.006	0.660	0.696	< 0.0001	< 0.0001
COPD	1.976	1.895	2.061	0.681	0.723	< 0.0001	< 0.0001
chronic liver disease	1.581	1.477	1.694	0.458	0.527	< 0.0001	< 0.0001
malignant neoplastic disease	1.235	1.198	1.273	0.211	0.241	< 0.0001	< 0.0001
HIV	1.825	1.514	2.197	0.601	0.787	< 0.0001	< 0.0001
history of transplant	1.268	1.017	1.581	0.238	0.458	0.805	1.000
stroke	2.125	2.014	2.243	0.754	0.808	< 0.0001	< 0.0001
opioid dependence	1.433	1.338	1.536	0.360	0.429	0.064	0.636
fully vaccinated	0.516	0.504	0.528	-0.662	0.639	< 0.0001	< 0.0001
boosted	0.467	0.454	0.479	-0.762	0.736	< 0.0001	< 0.0001
asthma	0.476	0.460	0.492	-0.743	0.710	< 0.0001	< 0.0001
inflammatory bowel disease	1.085	0.981	1.200	0.082	0.182	0.294	1.000
rheumatoid arthritis	1.241	1.143	1.347	0.216	0.298	0.006	0.067
multiple sclerosis	1.314	1.149	1.504	0.273	0.408	0.100	0.956
psoriatic arthritis	0.651	0.543	0.780	-0.429	0.249	0.001	0.013
psoriasis	0.715	0.656	0.778	-0.336	0.251	< 0.0001	< 0.0001
systemic sclerosis	1.064	0.776	1.459	0.062	0.378	0.681	1.000
spondyloarthritis	0.485	0.437	0.539	-0.723	0.618	< 0.0001	< 0.0001
systemic lupus	0.944	0.826	1.079	-0.057	0.076	0.005	0.061
vasculitis	1.391	1.107	1.745	0.330	0.557	0.473	1.000
sarcoidosis	0.769	0.622	0.950	-0.263	0.051	0.124	1.000
antiphospholipid syndrome	0.523	0.389	0.703	-0.648	0.352	0.007	0.075
Sjögren’s syndrome	0.916	0.760	1.104	-0.088	0.099	0.537	1.000
hydroxychloroquine	0.515	0.423	0.628	-0.663	0.465	0.006	0.067
methotrexate	1.087	0.888	1.330	0.083	0.285	0.845	1.000
leflunomide teriflunomide	0.673	0.446	1.017	-0.396	0.017	0.397	1.000
5-ASA	1.194	0.915	1.559	0.177	0.444	0.655	1.000
azathioprine	1.431	0.999	2.048	0.358	0.717	0.953	1.000
mercaptopurine	0.750	0.298	1.891	-0.287	0.637	0.386	1.000
mycophenolate	0.757	0.465	1.234	-0.278	0.210	0.490	1.000
calcineurin inhibitor	0.984	0.783	1.235	-0.017	0.211	0.170	1.000
TNF-α inhibitor	0.497	0.369	0.670	-0.699	0.401	0.025	0.258
fumarates	0.889	0.347	2.277	-0.118	0.823	0.946	1.000
interferons	0.930	0.094	9.152	-0.073	2.214	0.978	1.000
alkylating agent	1.269	0.540	2.986	0.239	1.094	0.672	1.000
hydroxyurea	0.948	0.564	1.594	-0.054	0.466	0.915	1.000
dapsone	1.103	0.566	2.149	0.098	0.765	0.593	1.000
IL-6 inhibitor	0.850	0.361	2.004	-0.163	0.695	0.857	1.000
IL-12/23 inhibitor	0.747	0.342	1.634	-0.291	0.491	0.577	1.000
IL-17 inhibitor	0.775	0.387	1.550	-0.256	0.438	0.693	1.000
IL-23 inhibitor	0.864	0.188	3.967	-0.146	1.378	0.916	1.000
abatacept	0.786	0.400	1.545	-0.241	0.435	0.780	1.000
S1P receptor modulator	0.951	0.397	2.280	-0.050	0.824	0.983	1.000
JAK inhibitor	1.262	0.795	2.002	0.232	0.694	0.873	1.000

PDE4i targeted synthetic	0.875	0.250	3.071	-0.133	1.122	0.935	1.000
anti-CD20	0.970	0.405	2.323	-0.030	0.843	0.988	1.000
budesonide	0.791	0.644	0.970	-0.235	0.030	0.211	1.000
systemic glucocorticoids	0.9	0.866	0.934	-0.106	0.068	< 0.0001	< 0.0001

Supplementary Table S4. Results of the multivariable logistic regression (LR) model, Selected factors for MV or death at Time Interval 1 (01/03/2020 to 25/12/2021) in COVID-19 positive patients.

Feature	Adjusted odds ratio (AOR)	95% confidence interval (low)	95% confidence interval (high)	Log of AOR	Feature importance	P value	P value (FDR corrected)
age	2.582	2.519	2.649	0.949	0.974	< 0.0001	< 0.0001
BMI	0.752	0.017	33.988	-0.285	3.526	0.887	1.000
sex: male	0.750	0.738	0.762	-0.288	0.272	< 0.0001	< 0.0001
hypertension	1.130	1.104	1.157	0.123	0.146	< 0.0001	< 0.0001
diabetes (type 1+2)	1.586	1.545	1.629	0.461	0.488	< 0.0001	< 0.0001
atrial fibrillation	1.859	1.793	1.927	0.620	0.656	< 0.0001	< 0.0001
coronary artery disease	1.247	1.203	1.293	0.221	0.257	< 0.0001	< 0.0001
heart failure	1.897	1.828	1.968	0.640	0.677	< 0.0001	< 0.0001
chronic kidney disease	2.139	2.069	2.210	0.760	0.793	< 0.0001	< 0.0001
COPD	2.043	1.964	2.125	0.715	0.754	< 0.0001	< 0.0001
chronic liver disease	1.492	1.398	1.590	0.400	0.464	0.002	0.026
malignant neoplastic disease	1.601	1.557	1.645	0.471	0.498	< 0.0001	< 0.0001
HIV	1.451	1.194	1.765	0.372	0.568	0.856	1.000
history of transplant	1.469	1.207	1.786	0.384	0.580	0.013	0.153
stroke	2.092	1.988	2.201	0.738	0.789	< 0.0001	< 0.0001
opioid dependence	1.012	0.946	1.082	0.012	0.079	< 0.0001	< 0.0001
fully vaccinated	0.145	0.142	0.149	-1.929	1.906	< 0.0001	< 0.0001
boosted	0.064	0.061	0.067	-2.752	2.704	< 0.0001	< 0.0001
asthma	0.428	0.414	0.442	-0.849	0.817	< 0.0001	< 0.0001
inflammatory bowel disease	0.657	0.593	0.729	-0.420	0.316	0.029	0.312
rheumatoid arthritis	1.122	1.038	1.213	0.115	0.193	0.969	1.000
multiple sclerosis	0.638	0.549	0.742	-0.449	0.298	0.005	0.061
psoriatic arthritis	0.808	0.677	0.965	-0.213	0.036	0.906	1.000
psoriasis	0.748	0.689	0.813	-0.290	0.207	< 0.0001	< 0.0001
systemic sclerosis	0.373	0.259	0.538	-0.986	0.620	0.100	0.890
spondyloarthritis	0.670	0.607	0.739	-0.401	0.302	0.001	0.014
systemic lupus	0.633	0.550	0.728	-0.458	0.317	0.001	0.014
vasculitis	2.654	2.113	3.333	0.976	1.204	0.233	1.000
sarcoidosis	1.005	0.829	1.219	0.005	0.198	0.313	1.000
antiphospholipid syndrome	0.891	0.677	1.172	-0.115	0.159	0.944	1.000
Sjögren's syndrome	0.344	0.277	0.429	-1.066	0.847	0.070	0.670
hydroxychloroquine	2.132	1.719	2.643	0.757	0.972	0.383	1.000
methotrexate	1.530	1.236	1.893	0.425	0.638	0.293	1.000
leflunomide teriflunomide	1.200	0.834	1.726	0.182	0.546	0.181	1.000

5-ASA	0.499	0.322	0.773	-0.695	0.258	0.212	1.000
azathioprine	1.604	1.074	2.396	0.473	0.874	0.417	1.000
mercaptopurine	0.772	0.333	1.790	-0.258	0.582	0.096	0.885
mycophenolate	1.603	0.944	2.721	0.472	1.001	0.967	1.000
calcineurin inhibitor	1.723	1.342	2.214	0.544	0.795	0.048	0.477
TNF-α inhibitor	0.543	0.399	0.739	-0.610	0.302	0.023	0.258
fumarates	2.244	1.256	4.011	0.808	1.389	0.486	1.000
interferons	0.974	0.030	31.817	-0.026	3.460	0.999	1.000
alkylating agent	1.342	0.589	3.059	0.294	1.118	0.825	1.000
hydroxyurea	0.699	0.365	1.338	-0.358	0.291	0.741	1.000
dapsone	1.389	0.749	2.573	0.328	0.945	0.673	1.000
IL-6 inhibitor	0.797	0.149	4.255	-0.227	1.448	0.619	1.000
IL-12/23 inhibitor	0.738	0.222	2.455	-0.304	0.898	0.826	1.000
IL-17 inhibitor	0.988	0.532	1.833	-0.012	0.606	0.796	1.000
IL-23 inhibitor	1.899	0.841	4.289	0.641	1.456	0.831	1.000
abatacept	0.807	0.215	3.031	-0.215	1.109	0.981	1.000
S1P receptor modulator	0.928	0.124	6.931	-0.075	1.936	0.985	1.000
JAK inhibitor	3.290	2.197	4.928	1.191	1.595	0.043	0.444
PDE4i targeted synthetic	0.783	0.232	2.646	-0.244	0.973	0.867	1.000
anti-CD20	1.281	0.616	2.664	0.247	0.980	0.845	1.000
budesonide	0.756	0.608	0.940	-0.279	0.062	0.659	1.000
systemic glucocorticoids	0.818	0.786	0.852	-0.200	0.160	< 0.0001	< 0.0001

Supplementary Table S5. Results of the multivariable logistic regression (LR) model, Selected factors for MV or death at Time Interval 2 (26/12/2021 to 30/08/2022) in COVID-19 positive patients.

Feature	Adjusted odds ratio (AOR)	95% confidence interval (low)	95% confidence interval (high)	Log of AOR	Feature importance	P value	P value (FDR corrected)
age	2.078	2.012	2.145	0.731	0.763	< 0.0001	< 0.0001
BMI	0.461	0.033	6.475	-0.774	1.868	0.392	1.000
sex: male	0.767	0.754	0.781	-0.265	0.247	< 0.0001	< 0.0001
hypertension	0.819	0.799	0.840	-0.200	0.174	< 0.0001	< 0.0001
diabetes (type 1+2)	1.238	1.202	1.274	0.213	0.242	0.003	0.043
atrial fibrillation	2.151	2.079	2.226	0.766	0.800	< 0.0001	< 0.0001
coronary artery disease	1.330	1.284	1.379	0.285	0.321	< 0.0001	< 0.0001
heart failure	2.563	2.474	2.654	0.941	0.976	< 0.0001	< 0.0001
chronic kidney disease	2.026	1.962	2.092	0.706	0.738	< 0.0001	< 0.0001
COPD	2.040	1.964	2.117	0.713	0.750	< 0.0001	< 0.0001
chronic liver disease	2.562	2.413	2.721	0.941	1.001	< 0.0001	< 0.0001
malignant neoplastic disease	1.856	1.806	1.908	0.619	0.646	< 0.0001	< 0.0001
HIV	1.272	1.069	1.513	0.241	0.414	0.271	1.000
history of transplant	1.821	1.495	2.217	0.599	0.796	0.806	1.000
stroke	2.166	2.067	2.270	0.773	0.820	< 0.0001	< 0.0001
opioid dependence	1.057	0.991	1.126	0.055	0.119	0.057	0.640
fully vaccinated	0.364	0.356	0.373	-1.010	0.986	< 0.0001	< 0.0001
boosted	0.306	0.298	0.314	-1.185	1.159	< 0.0001	< 0.0001

asthma	0.424	0.410	0.439	-0.857	0.823	< 0.0001	< 0.0001
inflammatory bowel disease	1.064	0.968	1.170	0.062	0.157	0.481	1.000
rheumatoid arthritis	1.156	1.068	1.251	0.145	0.224	0.594	1.000
multiple sclerosis	1.548	1.370	1.751	0.437	0.560	0.397	1.000
psoriatic arthritis	0.390	0.319	0.477	-0.941	0.740	0.062	0.667
psoriasis	0.802	0.738	0.871	-0.221	0.138	0.003	0.043
systemic sclerosis	0.581	0.405	0.834	-0.543	0.182	0.428	1.000
spondyloarthritis	0.392	0.353	0.435	-0.936	0.832	0.047	0.552
systemic lupus	0.763	0.668	0.871	-0.271	0.138	0.001	0.016
vasculitis	1.989	1.649	2.399	0.688	0.875	0.749	1.000
sarcoidosis	0.969	0.796	1.181	-0.031	0.166	0.924	1.000
antiphospholipid syndrome	0.901	0.697	1.165	-0.104	0.153	0.217	1.000
Sjögren's syndrome	0.807	0.665	0.981	-0.214	0.019	0.089	0.851
hydroxychloroquine	1.036	0.870	1.232	0.035	0.209	0.005	0.068
methotrexate	1.039	0.859	1.256	0.038	0.228	0.335	1.000
leflunomide teriflunomide	0.512	0.332	0.791	-0.670	0.235	0.412	1.000
5-ASA	0.982	0.753	1.281	-0.018	0.248	0.025	0.323
azathioprine	1.323	0.918	1.908	0.280	0.646	0.468	1.000
mercaptopurine	0.713	0.354	1.438	-0.338	0.363	0.079	0.785
mycophenolate	0.574	0.354	0.931	-0.555	0.071	0.815	1.000
calcineurin inhibitor	0.562	0.452	0.699	-0.576	0.358	0.078	0.785
TNF- α inhibitor	0.805	0.603	1.075	-0.217	0.072	0.038	0.467
fumarates	0.587	0.190	1.820	-0.532	0.599	0.724	1.000
interferons	0.856	0.113	6.488	-0.155	1.870	0.997	1.000
alkylating agent	1.131	0.502	2.550	0.123	0.936	0.732	1.000
hydroxyurea	1.240	0.823	1.868	0.215	0.625	0.630	1.000
dapsone	3.975	2.340	6.753	1.380	1.910	0.223	1.000
IL-6 inhibitor	1.407	0.754	2.627	0.342	0.966	0.705	1.000
IL-12/23 inhibitor	0.527	0.194	1.429	-0.641	0.357	0.668	1.000
IL-17 inhibitor	0.566	0.200	1.602	-0.570	0.471	0.695	1.000
IL-23 inhibitor	0.843	0.159	4.473	-0.171	1.498	0.981	1.000
abatacept	1.371	0.805	2.335	0.316	0.848	0.778	1.000
S1P receptor modulator	1.735	0.908	3.317	0.551	1.199	0.694	1.000
JAK inhibitor	0.533	0.323	0.877	-0.630	0.131	0.862	1.000
PDE4i targeted synthetic	0.809	0.182	3.593	-0.211	1.279	0.984	1.000
anti-CD20	0.568	0.185	1.737	-0.566	0.552	0.915	1.000
budesonide	0.483	0.389	0.599	-0.728	0.512	0.162	1.000
systemic glucocorticoids	0.921	0.889	0.954	-0.083	0.047	< 0.001	< 0.001

Supplementary Table S6. Results of the multivariable logistic regression (LR) model, Selected factors for death at Time Interval 1 (01/03/2020 to 25/12/2021) in COVID-19 positive patients.

Feature	Adjusted odds ratio (AOR)	95% confidence interval (low)	95% confidence interval (high)	Log of AOR	Feature importance	P value	P value (FDR corrected)
age	2.951	2.878	3.025	1.082	1.107	< 0.0001	< 0.0001
BMI	0.786	0.011	56.374	-0.240	4.032	0.919	1.000

sex: male	0.642	0.631	0.653	-0.443	0.426	< 0.0001	< 0.0001
hypertension	1.115	1.089	1.142	0.109	0.133	< 0.0001	< 0.0001
diabetes (type 1+2)	1.398	1.359	1.438	0.335	0.363	< 0.0001	< 0.0001
atrial fibrillation	1.996	1.923	2.073	0.691	0.729	< 0.0001	< 0.0001
coronary artery disease	1.527	1.471	1.584	0.423	0.460	< 0.0001	< 0.0001
heart failure	1.865	1.793	1.939	0.623	0.662	< 0.0001	< 0.0001
chronic kidney disease	2.825	2.729	2.924	1.039	1.073	< 0.0001	< 0.0001
COPD	2.378	2.282	2.479	0.866	0.908	< 0.0001	< 0.0001
chronic liver disease	1.449	1.354	1.551	0.371	0.439	0.002	0.027
malignant neoplastic disease	1.783	1.732	1.837	0.579	0.608	< 0.0001	< 0.0001
HIV	1.221	0.997	1.493	0.199	0.401	0.605	1.000
history of transplant	1.654	1.326	2.063	0.503	0.724	0.017	0.200
stroke	1.892	1.793	1.996	0.638	0.691	< 0.0001	< 0.0001
opioid dependence	0.968	0.902	1.038	-0.033	0.037	< 0.0001	< 0.0001
fully vaccinated	0.082	0.080	0.085	-2.498	2.470	< 0.0001	< 0.0001
boosted	0.012	0.011	0.013	-4.415	4.329	< 0.0001	< 0.0001
asthma	0.395	0.383	0.409	-0.928	0.895	< 0.0001	< 0.0001
inflammatory bowel disease	0.684	0.614	0.763	-0.379	0.271	0.077	0.795
rheumatoid arthritis	1.191	1.097	1.292	0.175	0.256	0.345	1.000
multiple sclerosis	0.549	0.465	0.648	-0.600	0.434	< 0.0001	< 0.0001
psoriatic arthritis	0.976	0.807	1.181	-0.024	0.166	0.542	1.000
psoriasis	0.584	0.534	0.640	-0.537	0.446	< 0.0001	< 0.0001
systemic sclerosis	0.508	0.349	0.740	-0.677	0.301	0.322	1.000
spondyloarthritis	0.778	0.703	0.861	-0.251	0.150	0.012	0.148
systemic lupus	0.693	0.602	0.799	-0.367	0.225	0.005	0.065
vasculitis	2.753	2.186	3.469	1.013	1.244	0.640	1.000
sarcoidosis	0.904	0.739	1.107	-0.101	0.102	0.873	1.000
antiphospholipid syndrome	1.078	0.815	1.426	0.075	0.355	0.748	1.000
Sjögren's syndrome	0.492	0.393	0.615	-0.710	0.486	0.346	1.000
hydroxychloroquine	2.142	1.721	2.667	0.762	0.981	0.664	1.000
methotrexate	1.028	0.796	1.326	0.027	0.282	0.641	1.000
leflunomide teriflunomide	1.003	0.654	1.537	0.003	0.430	0.373	1.000
5-ASA	0.910	0.631	1.314	-0.094	0.273	0.538	1.000
azathioprine	0.790	0.493	1.267	-0.235	0.237	0.896	1.000
mercaptopurine	0.851	0.383	1.895	-0.161	0.639	0.199	1.000
mycophenolate	0.948	0.524	1.716	-0.053	0.540	0.793	1.000
calcineurin inhibitor	4.043	3.043	5.371	1.397	1.681	0.020	0.225
TNF- α inhibitor	0.829	0.600	1.145	-0.188	0.135	0.076	0.795
fumarates	1.423	0.733	2.765	0.353	1.017	0.847	1.000
interferons	0.980	0.023	42.692	-0.020	3.754	1.000	1.000
alkylating agent	1.253	0.524	2.998	0.226	1.098	0.826	1.000
hydroxyurea	0.893	0.513	1.553	-0.114	0.440	0.345	1.000
dapsone	1.416	0.771	2.601	0.348	0.956	0.443	1.000
IL-6 inhibitor	0.815	0.133	5.013	-0.205	1.612	0.622	1.000
IL-12/23 inhibitor	0.798	0.182	3.501	-0.226	1.253	0.900	1.000
IL-17 inhibitor	1.208	0.646	2.259	0.189	0.815	0.899	1.000
IL-23 inhibitor	0.880	0.160	4.840	-0.128	1.577	0.984	1.000
abatacept	0.854	0.168	4.349	-0.157	1.470	0.989	1.000

S1P receptor modulator	0.931	0.122	7.092	-0.072	1.959	0.973	1.000
JAK inhibitor	2.283	1.501	3.473	0.826	1.245	0.109	1.000
PDE4i targeted synthetic	0.874	0.187	4.092	-0.134	1.409	0.968	1.000
anti-CD20	1.444	0.723	2.881	0.367	1.058	0.837	1.000
budesonide	0.948	0.751	1.197	-0.054	0.180	0.314	1.000
systemic glucocorticoids	0.934	0.897	0.974	-0.068	0.026	< 0.001	< 0.001

Supplementary Table S7. Results of the multivariable logistic regression (LR) model, Selected factors for death at Time Interval 2 (26/12/2021 to 30/08/2022) in COVID-19 positive patients.

Feature	Adjusted odds ratio (AOR)	95% confidence interval (low)	95% confidence interval (high)	Log of AOR	Feature importance	P value	P value (FDR corrected)
age	2.474	2.396	2.555	0.906	0.938	< 0.0001	< 0.0001
BMI	0.563	0.042	7.614	-0.575	2.030	0.384	1.000
sex: male	0.646	0.634	0.658	-0.437	0.418	< 0.0001	< 0.0001
hypertension	0.883	0.861	0.906	-0.125	0.099	< 0.0001	< 0.0001
diabetes (type 1+2)	1.085	1.053	1.117	0.081	0.111	0.244	1.000
atrial fibrillation	2.251	2.175	2.330	0.812	0.846	< 0.0001	< 0.0001
coronary artery disease	1.537	1.484	1.594	0.430	0.466	< 0.0001	< 0.0001
heart failure	2.479	2.392	2.568	0.908	0.943	< 0.0001	< 0.0001
chronic kidney disease	2.223	2.151	2.296	0.799	0.831	< 0.0001	< 0.0001
COPD	2.009	1.935	2.088	0.698	0.736	< 0.0001	< 0.0001
chronic liver disease	2.796	2.635	2.968	1.028	1.088	< 0.0001	< 0.0001
malignant neoplastic disease	2.084	2.026	2.143	0.734	0.762	< 0.0001	< 0.0001
HIV	1.634	1.381	1.933	0.491	0.659	0.130	1.000
history of transplant	2.031	1.674	2.465	0.709	0.902	0.337	1.000
stroke	1.870	1.784	1.960	0.626	0.673	< 0.0001	< 0.0001
opioid dependence	0.946	0.883	1.012	-0.056	0.012	0.001	0.016
fully vaccinated	0.301	0.294	0.308	-1.201	1.177	< 0.0001	< 0.0001
boosted	0.286	0.278	0.293	-1.253	1.227	< 0.0001	< 0.0001
asthma	0.331	0.319	0.343	-1.106	1.070	< 0.0001	< 0.0001
inflammatory bowel disease	1.078	0.983	1.183	0.076	0.168	0.905	1.000
rheumatoid arthritis	1.316	1.218	1.422	0.275	0.352	0.273	1.000
multiple sclerosis	0.923	0.805	1.060	-0.080	0.058	0.526	1.000
psoriatic arthritis	0.519	0.432	0.623	-0.657	0.474	0.084	0.803
psoriasis	0.647	0.593	0.705	-0.436	0.350	0.003	0.046
systemic sclerosis	0.746	0.535	1.040	-0.293	0.039	0.381	1.000
spondyloarthritis	0.446	0.401	0.496	-0.807	0.701	0.054	0.581
systemic lupus	0.872	0.766	0.993	-0.137	0.007	0.011	0.150
vasculitis	2.640	2.181	3.193	0.971	1.161	0.927	1.000
sarcoidosis	1.395	1.155	1.685	0.333	0.522	0.440	1.000
antiphospholipid syndrome	0.746	0.578	0.964	-0.293	0.037	0.096	0.885
Sjögren's syndrome	0.607	0.500	0.738	-0.499	0.304	0.022	0.271
hydroxychloroquine	1.401	1.183	1.659	0.337	0.506	0.004	0.057
methotrexate	0.911	0.750	1.107	-0.093	0.102	0.200	1.000

leflunomide teriflunomide	0.502	0.332	0.758	-0.690	0.277	0.472	1.000
5-ASA	0.992	0.760	1.296	-0.008	0.259	0.013	0.168
azathioprine	1.417	0.998	2.012	0.348	0.699	0.384	1.000
mercaptopurine	0.732	0.352	1.527	-0.311	0.423	0.039	0.458
mycophenolate	0.553	0.335	0.912	-0.593	0.092	0.805	1.000
calcineurin inhibitor	0.351	0.279	0.441	-1.047	0.819	0.075	0.745
TNF- α inhibitor	0.874	0.664	1.151	-0.134	0.141	0.049	0.550
fumarates	0.780	0.207	2.942	-0.249	1.079	0.802	1.000
interferons	0.923	0.113	7.561	-0.081	2.023	0.999	1.000
alkylating agent	1.080	0.502	2.326	0.077	0.844	0.654	1.000
hydroxyurea	1.655	1.124	2.438	0.504	0.891	0.495	1.000
dapsone	3.786	2.333	6.147	1.331	1.816	0.072	0.744
IL-6 inhibitor	1.359	0.717	2.573	0.307	0.945	0.617	1.000
IL-12/23 inhibitor	0.627	0.241	1.631	-0.467	0.489	0.640	1.000
IL-17 inhibitor	0.650	0.243	1.740	-0.431	0.554	0.730	1.000
IL-23 inhibitor	0.901	0.160	5.068	-0.104	1.623	0.987	1.000
abatacept	1.460	0.901	2.363	0.378	0.860	0.708	1.000
S1P receptor modulator	3.246	1.944	5.419	1.178	1.690	0.587	1.000
JAK inhibitor	0.629	0.381	1.040	-0.464	0.039	0.704	1.000
PDE4i targeted synthetic	0.939	0.345	2.560	-0.063	0.940	0.774	1.000
anti-CD20	0.699	0.218	2.246	-0.358	0.809	0.947	1.000
budesonide	0.593	0.485	0.725	-0.522	0.321	0.257	1.000
systemic glucocorticoids	1.020	0.984	1.058	0.020	0.056	< 0.0001	< 0.0001

Supplementary Table S8. SNOMED definitions of IMIDs in this paper.

The SNOMED Concept IDs used to define certain types of IMIDs in the research paper.

IMID type	SNOMED Concept ID
asthma	266361008, 426656000, 405944004, 10676431000119103, 10742121000119104, 708038006, 233678006, 56968009, 2360001000004109, 125011000119100, 195949008, 1086701000000102, 34015007, 10675991000119100, 829976001, 16584951000119101, 782513000, 786836003, 370220003, 735588005, 703953004, 135171000119106, 734905008, 10692681000119108, 1086711000000100, 418395004, 708093000, 10674711000119105, 733858005, 707445000, 401000119107, 708090002, 93432008, 72301000119103, 233683003, 304527002, 424643009, 707981009, 424199006, 10675551000119104, 10674991000119104, 404808000, 407674008, 370218001, 707513007, 404806001, 901000119100, 782520007, 10675391000119101, 703954005, 63088003, 233688007, 1103911000000103, 12428000, 735589002, 1751000119100, 708094006, 389145006, 10675911000119109, 233679003, 16055311000119107, 707512002, 10676391000119108, 707447008, 425969006, 92807009, 10675751000119107, 707979007, 370219009, 18041002, 10692761000119107, 10675871000119106, 708096008, 225057002, 125021000119107, 707444001, 195967001, 866881000000101, 59786004, 442025000, 10692721000119102, 1741000119102, 19849005, 427679007, 707980005, 404804003, 423889005, 31387002, 762521001, 11641008, 370221004, 10675471000119109, 734904007, 55570000, 124991000119109, 708095007, 707511009, 57607007, 281239006, 41553006, 195977004, 409663006, 37981002, 445427006, 427295004, 103781000119103, 10675431000119106, 426979002, 99031000119107, 233687002, 427603009, 10676511000119109, 5281000124103, 135181000119109, 707446004, 125001000119103
inflammatory bowel disease	'295046003', '733157003', '721686000', '128600008', '24829000', '56689002', '71833008', '52506002', '3815005', '50440006', '1085131000119105', '426549001', '737195007', '410485009', '414156000', '61424003', '239814006', '1085901000119101', '235664007', '8161000119106', '444546002', '235714007', '1085231000119100', '201727001', '34000006', '10743231000119101', '697969008', '24526004', '1092841000119100', '1085801000119106', '15342002', '14311001', '414153008', '397173003', '414154002', '201807008', '91390005', '56287005', '239809007', '201805000', '1085851000119105', '196987008', '732966008', '235607002', '13470001', '196977009', '70622003', '1085751000119100', '78324009', '397172008', '444548001', '722850002', '7620006',

	'196578009', '445243001', '441971007', '64766004', '442159003', '234999001', '788718000', '38106008', '78712000', '721702009', '410484008'
rheumatoid arthritis	'1073741000119109', '239792003', '11055151000119108', '193180002', '402433007', '1073771000119102', '201772009', '459911000124100', '239804002', '193250002', '735599007', '319031000119108', '1073681000119109', '1073781000119104', '201766009', '201784006', '201780002', '1073791000119101', '410798004', '319841000119107', '201774005', '287008006', '239803008', '201785007', '201768005', '410802003', '201771002', '287006005', '410801005', '38877003', '201778008', '143441000119108', '59165007', '28880005', '15691761000119107', '201783000', '427770001', '399923009', '1073691000119107', '1073811000119102', '15686321000119106', '318941000119109', '239791005', '1073831000119107', '15687841000119108', '86219005', '239795001', '201769002', '201773004', '410796000', '318951000119106', '239799007', '735600005', '15686001000119104', '52661003', '201770001', '15687201000119107', '318871000119107', '410800006', '15685921000119102', '410502007', '15685961000119107', '201779000', '1073711000119105', '201767000', '201777003', '7607008', '319081000119109', '781206002', '77522006', '201775006', '123803006', '62131000000107', '201791009', '15686281000119101', '1073761000119108', '201764007', '1073721000119103', '16024431000119108', '15691161000119108', '410797009', '287007001', '16044751000119106', '239802003', '239941000', '1073731000119100', '57160007', '429192004', '15691801000119104', '15687321000119109', '201776007', '1073821000119109', '15691721000119102', '1073701000119107', '1073751000119106', '300961000119108', '301051000119101', '308143008', '1073801000119100', '398640008', '400054000', '318881000119105', '459921000124108', '410799007', '69896004', '201781003', '319111000119104', '129563009', '201796004'
multiple sclerosis	'703621006', '230372003', '439567002', '724778008', '816984002', '426373005', '425500002', '445967004', '24700007', '903741000000102', '733028000', '434321000124107', '428700003', '192929006', '766246000', '192927008', '438511000', '192926004', '703622004'
psoriatic arthritis	'239803008', '10629311000119107', '200956002', '239813000', '239802003', '239804002', '410482007', '156370009', '239812005'
psoriasis	'238600001', '200969003', '402316001', '402314003', '238616004', '402319008', '402317005', '402326008', '402311006', '402333008', '238611009', '200966005', '402308005', '402336000', '402331005', '238605006', '402330006', '784327005', '238608008', '702617007', '200965009', '200962007', '3533007', '27520001', '200967001', '111188005', '402309002', '402324006', '402318000', '238601002', '402307000', '200968006', '402320002', '28840001', '81271001', '238617008', '238603004', '200975007', '200973000', '238602009', '200972005', '238609000', '200970002', '402312004', '37042000', '238612002', '200971003', '200977004', '239813000', '402315002', '200974006', '238606007', '83839005', '402313009', '402334002', '9014002', '721538000', '33339001', '200963002', '402322005', '402332003', '402323000', '402327004', '200964008', '402325007', '19514005', '238613007', '784339002', '402335001', '402310007', '402321003', '402329001', '719810000', '65539006', '239098009', '238604005', '784328000', '400069004', '402337009', '25847004', '238615000'
systemic sclerosis	'196133001', '322461000119108', '236502006', '403519001', '298285004', '236503001', '443872005', '403517004', '402712002', '724603009', '193252005', '774080007', '403520007', '403516008', '715401008', '444133002', '201443009', '22784002', '128461001', '403518009', '87442008', '403514006', '402713007', '35719004', '62382002', '51156002', '89681000119101', '89155008', '299276009', '403515007', '128460000', '31848007'
spondyloarthritis	'239806000', '67224007', '239808004', '724606001', '239810002', '235066005', '200956002', '236744002', '234524009', '9350004', '402339007', '162930007', '201776007', '870281008', '1073391000119100', '786077003', '239805001', '328211000119107', '201568008', '723116002', '239813000', '55146009', '1073381000119103', '264516005', '15630971000119102', '10317009', '239815007', '784332006', '713777005', '239811003', '710813008', '15972981000119101', '1074981000119100', '201736002', '9631008', '49807009', '402338004', '239812005', '431236003', '1074971000119103', '371104006', '110041000119104', '15690361000119104', '238407005', '156370009'
systemic lupus erythematosus	'25380002', '54912002', '773333003', '309762007', '724767000', '19682006', '402711009', '724781003', '239888002', '397856003', '200936003', '61458006', '403487009', '80258006', '403486000', '77753005', '238652000', '201436003', '698694005', '4676006', '239886003', '196138005', '68815009', '55464009', '54072008', '239889005', '713225000', '239890001', '76521009', '36402006', '200940007', '239887007', '11013005', '73286009', '295121000119101', '22888007', '95609003', '52042003', '707301001', '295101000119105', '95644001', '403488004', '295111000119108'
uveitis	'418839003', '818950005', '352941000119102', '416666007', '786077003', '726078000', '699861000', '312930008', '415359008', '410481000', '86219005'
vasculitis	'988111000000106', '239926000', '724599009', '359789008', '724502006', '239929007', '128971000119101', '239936008', '402662002', '53485006', '239930002', '232460001', '38675009', '11352009', '58144006', '722020006', '239946005', '28807005', '190815001', '30911005', '57484009', '724783000', '75053002', '191306005', '3275009', '722191003', '310701003', '21542005', '718217000', '195353004', '724600007', '239935007', '239947001', '232369001', '155441006',

	'239927009', '54034003', '235000001', '44371002', '239922003', '403443000', '57390009', '239938009', '723674005', '402856005', '82275008', '239939001', '239945009', '762302008', '230732009', '239925001', '239921005', '239928004'
sarcoidosis	'724780002', '402369000', '58870009', '735433009', '697921005', '75403004', '64757003', '233743002', '233744008', '238676008', '402371000', '238680003', '91259005', '402370004', '55941000', '234528007', '54515008', '234524009', '233771008', '707238003', '197368002', '193195000', '232368009', '402372007', '238678009', '111937006', '233769008', '111292008', '195033009', '402380000', '238679001', '9529007', '24369008', '193251003', '37061001', '192673008', '402368008', '233770009', '361197009', '402373002', '231799005', '234526006', '233767005', '72470008', '230193008', '361198004', '400127001', '31541009', '232458003', '233768000', '21787007', '238677004', '234529004', '111936002', '233772001', '234530009', '870334009', '4416007', '234531008', '425384007', '310607007', '80941006', '238674006', '17363001', '234527002', '187233002', '238681004', '402379003', '17919002'
antiphospholipid syndrome	'239895006', '239894005', '774084003', '239897003', '239892009', '72161000119100', '402865003', '19267009', '26843008', '609329007'
Sjögren's syndrome	'196137000', '239915006', '762303003', '126766000', '83901003', '78946008', '724782005', '239912009'

Supplementary Table S9. Specific medications of each immunomodulatory drug class and monoclonal antibody class in this paper.

The list of actual medications used to define a certain class of immunomodulatory drug in the research paper. Only included the following routes of those medications: 'Oral', 'Intramuscular', 'Intravenous', 'Subcutaneous Infusion', 'Subcutaneous', 'Intravenous (Continuous Infusion)', 'Rectal'.

Drug class	Example of medications and ingredients	Ingredients RxNorm codes
hydroxychloroquine	hydroxychloroquine	5521
methotrexate	methotrexate	6851
Leflunomide,<br>teriflunomide	leflunomide,<br>teriflunomide	27169, 1310520
5-ASA	balsalazide, sulfaSALAzine, mesalamine	18747, 9524, 52582
azathioprine	azathioprine	1256
mercaptopurine	mercaptopurine	103
mitoxantrone	mitoxantrone	7005
mycophenolate	mycophenolate	265323
calcineurin inhibitor	cycloSPORINE, sirolimus, tacrolimus	3008, 35302, 42316
TNF-α inhibitor	adalimumab, certolizumab pegol, etanercept, golimumab, inFLIXimab	327361, 709271, 214555, 819300, 191831
fumarates	dimethyl fumarate, diroximel fumarate, monomethyl fumarate	1373478, 2261783, 1546433
interferons	interferon beta-1a, interferon beta-1b	75917, 72257
alkylating agent	chlorambucil	2346
hydroxyurea	hydroxyurea	5552
dapsone	dapsone	3108
cladribine	cladribine	44157
IL-1 inhibitor	canakinumab, anakinra, rilonacept	853491, 72435, 763450
IL-6 inhibitor	sarilumab, tocilizumab, satralizumab	1923319, 612865, 2391541
IL-12/23 inhibitor	ustekinumab	847083
IL-17 inhibitor	ixekizumab, brodalumab, secukinumab	1745099, 1872251, 1599788
IL-23 inhibitor	guselkumab, tildrakizumab, risankizumab	1928588, 2053436, 2166040
abatacept	abatacept	614391
anti-BLyS	belimumab	1092437
S1P receptor modulator	siponimod, ponesimod, fingolimod, ozanimod	2121085, 2532300, 1012892, 2288236
JAK inhibitor	tofacitinib, upadacitinib, baricitinib	1357536, 2196092, 2047232
Integrin inhibitor	vedolizumab, natalizumab	1538097, 354770

PDE4 inhibitor	apremilast	1492727
anti-CD20	riTUXimab, ocrelizumab, ofatumumab	121191, 1876366, 712566
anti-CD52	alemtuzumab	117055
budesonide	budesonide	19831
systemic glucocorticoids	predniSONE, dexamethasone, prednisoLONE, triamcinolone, methylPREDNISolone, hydrocortisone	8638, 3264, 8638, 10759, 6902, 5492
Anti-SARS-CoV-2 monoclonal antibody	bamlanivimab, bebelove mab, casirivimab, cilgavimab, etesevimab, etesevimab, imdevimab, sotrovimab, tixagevimab	2463114, 2592360, 2465242, 2587306, 2477854, 2465249, 2550731, 2587300
nirmatrelvir–ritonavir	nirmatrelvir, lopinavir/ritonavir	284640, 2587899, 2599543

Supplementary Table S10. TRIPOD Statement.

A checklist of items in accordance with TRIPOD reporting guidelines.[1] This includes the page numbers in the manuscript in which the information that fulfils each item on the checklist can be found.

Section/Topic			Checklist Item	Page
**Title and abstract**				
Title	1	D;V	Identify the study as developing and/or validating a multivariable prediction model, the target population, and the outcome to be predicted.	1
Abstract	2	D;V	Provide a summary of objectives, study design, setting, participants, sample size, predictors, outcome, statistical analysis, results, and conclusions.	1
**Introduction**				
Background and objectives	3a	D;V	Explain the medical context (including whether diagnostic or prognostic) and rationale for developing or validating the multivariable prediction model, including references to existing models.	2
	3b	D;V	Specify the objectives, including whether the study describes the development or validation of the model or both.	3-10
**Methods**				
Source of data	4a	D;V	Describe the study design or source of data (e.g., randomized trial, cohort, or registry data), separately for the development and validation data sets, if applicable.	3-4; Figure 1
	4b	D;V	Specify the key study dates, including start of accrual; end of accrual; and, if applicable, end of follow-up.	3-4; Figure 1-2
Participants	5a	D;V	Specify key elements of the study setting (e.g., primary care, secondary care, general population) including number and location of centres.	3-4
	5b	D;V	Describe eligibility criteria for participants.	4; Figure 1
	5c	D;V	Give details of treatments received, if relevant.	5-9; Table 2
Outcome	6a	D;V	Clearly define the outcome that is predicted by the prediction model, including how and when assessed.	3

	6b	D;V	Report any actions to blind assessment of the outcome to be predicted.	3-4
Predictors	7a	D;V	Clearly define all predictors used in developing or validating the multivariable prediction model, including how and when they were measured.	5-9; Table 2
	7b	D;V	Report any actions to blind assessment of predictors for the outcome and other predictors.	3-4
Sample size	8	D;V	Explain how the study size was arrived at.	4; Figure 1
Missing data	9	D;V	Describe how missing data were handled (e.g., complete-case analysis, single imputation, multiple imputation) with details of any imputation method.	Table 2
Statistical analysis methods	10a	D	Describe how predictors were handled in the analyses.	3
	10b	D	Specify type of model, all model-building procedures (including any predictor selection), and method for internal validation.	3-4
	10c	V	For validation, describe how the predictions were calculated.	4; Figure 2
	10d	D;V	Specify all measures used to assess model performance and, if relevant, to compare multiple models.	3-4; Table S1
	10e	V	Describe any model updating (e.g., recalibration) arising from the validation, if done.	3-4; Table S1
Risk groups	11	D;V	Provide details on how risk groups were created, if done.	NA
Development vs. validation	12	V	For validation, identify any differences from the development data in setting, eligibility criteria, outcome, and predictors.	3-4
**Results**				
Participants	13a	D;V	Describe the flow of participants through the study, including the number of participants with and without the outcome and, if applicable, a summary of the follow-up time. A diagram may be helpful.	4; Figure 1
	13b	D;V	Describe the characteristics of the participants (basic demographics, clinical features, available predictors), including the number of participants with missing data for predictors and outcome.	5-9; Table 2
	13c	V	For validation, show a comparison with the development data of the distribution of important variables (demographics, predictors and outcome).	5-9; Table 1-2
Model development	14a	D	Specify the number of participants and outcome events in each analysis.	5; Table 1
	14b	D	If done, report the unadjusted association between each candidate predictor and outcome.	N/A
Model specification	15a	D	Present the full prediction model to allow predictions for individuals (i.e., all regression coefficients, and model intercept or baseline survival at a given time point).	N/A
	15b	D	Explain how to the use the prediction model.	N/A

Model performance	16	D;V	Report performance measures (with CIs) for the prediction model.	11; Table S3-S8
Model-updating	17	V	If done, report the results from any model updating (i.e., model specification, model performance).	Figure S1
**Discussion**				
Limitations	18	D;V	Discuss any limitations of the study (such as nonrepresentative sample, few events per predictor, missing data).	16
Interpretation	19a	V	For validation, discuss the results with reference to performance in the development data, and any other validation data.	12-16
	19b	D;V	Give an overall interpretation of the results, considering objectives, limitations, results from similar studies, and other relevant evidence.	12-16
Implications	20	D;V	Discuss the potential clinical use of the model and implications for future research.	16
**Other information**				
Supplementary information	21	D;V	Provide information about the availability of supplementary resources, such as study protocol, Web calculator, and data sets.	Y
Funding	22	D;V	Give the source of funding and the role of the funders for the present study.	17

Supplementary Table S11. STROBE Statement.

A checklist of items in accordance with STROBE reporting guidelines.[2] This includes the page numbers in the manuscript in which the information that fulfils each item on the checklist can be found.

	Item No·	Recommendation	Page No·	Relevant text from manuscript
**Title and abstract**	1	(*a*) Indicate the study's design with a commonly used term in the title or the abstract	1	Risk factors for severe COVID-19 outcomes: a study of immune-mediated inflammatory diseases, immunomodulatory medications, and comorbidities in a large US healthcare system; Title
		(*b*) Provide in the abstract an informative and balanced summary of what was done and what was found	1	Abstract
**Introduction**				

Background/rationale	2	Explain the scientific background and rationale for the investigation being reported	2	Introduction
Objectives	3	State specific objectives, including any prespecified hypotheses	2	Here, we analyse the risk for severe COVID-19 outcomes, using multivariable models across a large US population to investigate how specific IMIDs, classes of immunomodulatory drugs, chronic comorbidities, and vaccination status are associated with risk for severe COVID-19 outcomes, and compare the pre-Omicron and Omicron-predominant time periods·
**Methods**				
Study design	4	Present key elements of study design early in the paper	2	Using multivariable models across a large US population
Setting	5	Describe the setting, locations, and relevant dates, including periods of recruitment, exposure, follow-up, and data collection	3-10	Method
Participants	6	(*a*) *Cohort study*—Give the eligibility criteria, and the sources and methods of selection of participants· Describe methods of follow-up<br>*Case-control study*—Give the eligibility criteria, and the sources and methods of case ascertainment and control selection· Give the rationale for the choice of cases and controls<br>*Cross-sectional study*—Give the eligibility criteria, and the sources and methods of selection of participants	3 and 4	*Study setting and participants; Cohort characteristics*
		(*b*) *Cohort study*—For matched studies, give matching criteria and number of exposed and unexposed<br>*Case-control study*—For matched studies, give matching criteria and the number of controls per case	N/A	This was an unmatched study
Variables	7	Clearly define all outcomes, exposures, predictors, potential confounders, and effect modifiers· Give diagnostic criteria, if applicable	3 and 4	*COVID-19 severe outcomes;* Figure 2
Data sources/ measurement	8*	For each variable of interest, give sources of data and details of methods of assessment (measurement)· Describe comparability of assessment methods if there is more than one group	4	Figure 2; Supplementary Table S9; Supplementary Table S10

Bias	9	Describe any efforts to address potential sources of bias	3-10	Models were trained for two SARS-CoV-2 positive cohorts (those infected before the pre-dominance of Omicron variants and those infected later)·Models were trained on 90% of the data with (over-sampling or over-weight on minority classes to address class imbalance in training data), with 10% of the data held out for independent performance testing·
Study size	10	Explain how the study size was arrived at	4	Figure 1
Quantitative variables	11	Explain how quantitative variables were handled in the analyses· If applicable, describe which groupings were chosen and why	3-5	*Machine learning models used for classification*
Statistical methods	12	(*a*) Describe all statistical methods, including those used to control for confounding	3-5	*Machine learning models used for classification*
		(*b*) Describe any methods used to examine subgroups and interactions	N/A	-
		(*c*) Explain how missing data were addressed	3	*Machine learning models used for classification*
		(*d*) *Cohort study*—If applicable, explain how loss to follow-up was addressed<br>*Case-control study*—If applicable, explain how matching of cases and controls was addressed<br>*Cross-sectional study*—If applicable, describe analytical methods taking account of sampling strategy	3	*Study setting and participants*
		(*e*) Describe any sensitivity analyses	10	*Machine learning models used for classification*
**Results**				

Participants	13*	(a) Report numbers of individuals at each stage of study—eg numbers potentially eligible, examined for eligibility, confirmed eligible, included in the study, completing follow-up, and analysed	3	*Study setting and participants*
		(b) Give reasons for non-participation at each stage	N/A	-
		(c) Consider use of a flow diagram	4	Figure 1
Descriptive data	14*	(a) Give characteristics of study participants (eg demographic, clinical, social) and information on exposures and potential confounders	5-9	Table 1 and Table 2
		(b) Indicate number of participants with missing data for each variable of interest	N/A	There was no missing data after imputation
		(c) *Cohort study*—Summarise follow-up time (eg, average and total amount)	N/A	This was a retrospective study
Outcome data	15*	*Cohort study*—Report numbers of outcome events or summary measures over time	5	Table 1
		Case-control study—Report numbers in each exposure category, or summary measures of exposure	N/A	This was a retrospective study
		Cross-sectional study—Report numbers of outcome events or summary measures	N/A	This was a retrospective study
Main results	16	(*a*) Give unadjusted estimates and, if applicable, confounder-adjusted estimates and their precision (eg, 95% confidence interval)· Make clear which confounders were adjusted for and why they were included	10-15	Results
		(*b*) Report category boundaries when continuous variables were categorized	3	Continuous variables were normalized by applying min-max transformation
		© If relevant, consider translating estimates of relative risk into absolute risk for a meaningful time period	N/A	There were no estimates of relative risk
Other analyses	17	Report other analyses done—eg analyses of subgroups and interactions, and sensitivity analyses	N/A	There were no subgroups

**Discussion**				
Key results	18	Summarise key results with reference to study objectives	12	Discussion
Limitations	19	Discuss limitations of the study, taking into account sources of potential bias or imprecision· Discuss both direction and magnitude of any potential bias	12	Discussion
Interpretation	20	Give a cautious overall interpretation of results considering objectives, limitations, multiplicity of analyses, results from similar studies, and other relevant evidence	12-16	Discussion
Generalisability	21	Discuss the generalisability (external validity) of the study results	16	Discussion; In strength and limitation paragraphs
**Other information**				
Funding	22	Give the source of funding and the role of the funders for the present study and, if applicable, for the original study on which the present article is based	1	Abstract

Supplementary Table S12. Classification Machine learning algorithms hyperparameters and hyperparameter values used in performance evaluation.

Model name	Name	Value
Logistic Regression	missing	('drop')
	method	('lbfgs')
K Nearest Neighbours	n_neighbors	(10)
	weights	('uniform')
	algorithm	('auto')
	leaf_size	(30)
	p	(2)
Support Vector Machine	kernel	('rbf')
	C	(1)
	gamma	('scale')
	shrinking	(True)
	probability	(True)
	class_weight	('balanced')
	random_state	(42)
Adaptive Boosting	n_estimators	(200)
	learning_rate	(0.05)
	algorithm	('SAMME.R')
	random_state	(42)

Supplementary Discussion

Supplementary Discussion S1. Anti-SARS-CoV-2 neutralizing monoclonal antibodies (NmAbs) analysis.

Anti-SARS-CoV-2 NmAbs were only administered to 0·41% (699 / 169 993) of patients with positive COVID-19 tests during the pre-Omicron period, and only 2·34% (2 822 / 120 862) during the Omicron pre-dominant period. (Table 2). NmAbs were significantly associated with reduced risk for severe outcomes in both periods. Both the NmAbs analysis and the analysis excluding patients who received NmAbs showed similar results to the main analyses for other variables. The additional analyses on NmAbs showed that few patients received this treatment. NmAbs had been recommended for patients who test positive and are considered at high risk but do not yet require hospitalisation. Because IMIDs and immunomodulatory medications have sometimes been assumed to confer high risk, the significant association between NmAbs and better outcomes may reflect a combination of treatment efficacy and confounding by indication.

Supplementary Discussion S2. Nirmatrelvir-lopinavir/ritonavir analysis.

Nirmatrelvir-lopinavir/ritonavir was only administered to 0·12% (146 / 120 862) of patients with positive COVID-19 tests during the Omicron pre-dominant period. (Table 2). Nirmatrelvir-lopinavir/ritonavir did not reach significance during the Omicron time period which it is studied.

Supplementary References

[1] von Elm E, Altman DG, Egger M, et al. STROBE Initiative. The Strengthening the Reporting of Observational Studies in Epidemiology (STROBE) statement: guidelines for reporting observational studies. Lancet. 2007 Oct 20;370(9596):1453-7.

[2] Collins GS, Reitsma JB, Altman DG, et al. Transparent reporting of a multivariable prediction model for individual prognosis or diagnosis (TRIPOD): the TRIPOD statement. BMJ. 2015 Jan 7;350:g7594.
